# Electrically tunable conducting oxide metasurfaces for high power applications

**DOI:** 10.1515/nanoph-2022-0594

**Published:** 2023-01-18

**Authors:** Ruzan Sokhoyan, Prachi Thureja, Jared Sisler, Meir Grajower, Komron Shayegan, Eyal Feigenbaum, Selim Elhadj, Harry A. Atwater

**Affiliations:** Thomas J. Watson Laboratories of Applied Physics, California Institute of Technology, Pasadena, CA 91125, USA; National Ignition Facility and Photon Science, Lawrence Livermore National Laboratory, Livermore, CA 94550, USA; Materials Engineering Division, Lawrence Livermore National Laboratory, Livermore, CA 94550, USA

**Keywords:** active metasurface, cadmium oxide, free space optical communications, high power illumination, indium tin oxide, LiDAR, thermal analysis

## Abstract

Active metasurfaces designed to operate at optical frequencies are flat optical elements that can dynamic, subwavelength-scale wavefront control of reflected or transmitted light. The practical and fundamental power-handling limits of active metasurfaces at high pulse energies and high average powers determine the potential applications for these emerging photonic components. Here, we investigate thermal performance limits of reflective gate-tunable conducting oxide metasurfaces illuminated with high power density laser beams, for both continuous wave (CW) and pulsed laser illumination. Our gate-tunable metasurfaces use indium tin oxide (ITO) as an active material, which undergoes an epsilon-near-zero (ENZ) transition under applied electrical bias. We experimentally show that under CW illumination, there is no significant change in the electrically tunable metasurface optical response for high irradiances ranging from 1.6 kW/cm^2^ to 9.1 kW/cm^2^ when the illuminating laser beam diameter is 7 μm. Even under an applied bias, when over 60% of the incoming light is absorbed in a 1 nm–thick charge accumulation layer within ITO, the local temperature rise in the metasurface is modest, supporting its robustness for high-power applications. Additionally, we theoretically show that in the ENZ regime, the metasurface reflectance can be increased by a factor of 10 by replacing the active ITO layer with cadmium oxide (CdO). Thus conducting oxide metasurfaces can tolerate the power densities needed in higher power applications, including free space optical communications, to light detection and ranging (LiDAR), as well as laser-based additive manufacturing.

## 1 Introduction

Metasurfaces are ultrathin arrays of optical scatterers, which can tailor the wavefront of the reflected or transmitted light at a subwavelength scale [1, 2]. They can perform optical functions such as lensing [3, 4] and polarization control [5] typically achieved by using bulky, non-flat optical components. Moreover, a single ultrathin metasurface can perform optical functions which otherwise are only attainable by combining multiple bulk optical components [6]. Thus, metasurfaces hold extraordinary promise for optical component miniaturization and further on-chip integration. The optical response of metasurfaces is typically controlled by changing the geometrical parameters of the subwavelength scatterers, also referred to as metasurface elements. As a result, the optical response of these geometry-controlled metasurfaces (referred to as passive metasurfaces) cannot be modified once the metasurfaces have been fabricated.

In recent years, a paradigm of active metasurfaces for real-time rapid control of the wavefront of light at a subwavelength scale has emerged [7]. In a prototypical active metasurface, the phase and amplitude of light scattered by each metasurface element can be dynamically and reversibly controlled via application of external stimuli such as voltage or heat. Previous works have exploited different physical mechanisms to actively control the properties of the light reflected from or transmitted through metasurfaces [8–26]. Active metasurfaces have been experimentally demonstrated by using quantum confined Stark effect in multiple quantum wells [8], phase transitions in germanium antimony telluride (GST) [9, 10, 27] and vanadium dioxide (VO_2_) [11, 12], ionic transport [13], reorientation of liquid crystal molecules [14, 15], mechanical deformations [16, 17], and field effect in indium tin oxide (ITO) [18–22], gallium arsenide [23], silicon [24], and graphene [25, 26]. Most of the prior experimental reports on active metasurfaces do not demonstrate the ability to actively control the phase imposed by individual metasurface elements. However, the active phase control of individual metasurface elements is necessary for realization of a versatile reconfigurable metasurface device capable of performing multiple different optical functions.

Recently, we experimentally demonstrated an actively reconfigurable *multifunctional* metasurface, which is capable of performing diverse optical functions [20]. This multifunctional metasurface, which can either steer or focus the reflected light depending on the configuration of an externally applied bias [20], operates in the near-infrared wavelength range and utilizes ITO as an active electronically controlled material. It has been previously shown that the phase of light reflected from ITO-based active metasurfaces can be continuously tuned with an applied bias voltage [18–21]. This active phase control, accompanied with addressability of individual metasurface elements, enables diverse wavefront control [20]. High modulation frequencies (∼10 MHz) [18] and low power consumption are another important advantage of ITO-based active metasurfaces relative to mechanically reconfigured optical components. Hence, gate-tunable ITO-based metasurfaces have potential to achieve a high degree of technological maturity.

Active metasurfaces could thus form the foundation of future high performance and low-cost chip-scale light detection and ranging (LiDAR) systems, which can be used in autonomous vehicles [28]. Commercially available LiDARs typically steer the beams via mechanical motion [29]. For example, autonomous vehicle LiDARs use mechanical rotation [30], Risley prisms [31], or micro-electro-mechanical motion [32] to steer the beam. However, these solutions result in bulky form factors and high cost of the resulting LiDAR systems. All-solid-state ultrafast beam steering units could miniaturize LiDARs and reduce their cost. Miniaturized beam steering units could also be used in free-space optical communications in which data is modulated onto C-band lasers (1530–1565 nm) to transmit information (for detailed link budget analysis see Supplementary Materials, Part 1). Compared to conventional radiofrequency antennas, future free-space optical communication systems could greatly increase spacecraft communications bandwidth while dramatically decreasing the required size, weight, and power (SWaP) [33]. We propose that active metasurfaces have the potential to be used for beam shaping of high-power (∼kW/cm^2^) industrial laser beams for machining or for additive manufacturing of metals [34]. Moreover, the manufacturing process can be significantly accelerated since electronically controlled metasurfaces, in principle, enable ultrafast beam manipulation (for an additional discussion see Supplementary Materials, Part 1). In summary, creating both low-profile and high-power handling chip-scale beam steering systems could be impactful for a number of technologies.

To provide some perspective, recent advances in chip-scale non-mechanical beam steering have been largely based either on metasurfaces or optical phased arrays [35, 36]. While steering beams via optical phased arrays is another active research area, there are a number of issues associated with this approach. A Si photonic chip architecture featuring arrays of waveguides coupled to phase shifters is limited in its total output power by the maximum tolerable power density of the input silicon waveguide. This limitation arises because of free carrier absorption of induced carriers, two photon absorption, and Kerr nonlinear optical response of Si, which are non-negligible at high optical powers [37]. To overcome the limitation caused by silicon waveguides, a SiN on Si platform has been proposed [38]. Using a SiN waveguide network, for an input power of 9.1 W, an output power of 400 mW has been achieved [38]. In the mentioned work [38], the area of the considered aperture was 0.4 cm × 0.4 cm, yielding an output power per unit area of 2.5 W/cm^2^. The ultimate limit of power handling capabilities of SiN on Si photonic chips is yet to be established. Moreover, most optical phased arrays utilize p-i-n diode phase shifting waveguides, which are relatively large. To achieve two-dimensional beam steering, optical phased arrays steer the beam in one axis using phase control and in the other axis using wavelength tuning. This deconvolution of the phase shifter and the optical emitter limits the versatility of optical phased arrays, and hence their usage in applications beyond beam steering poses significant challenges. In the case of metasurfaces, the phase shifter and optical emitter are combined in a single optical component metasurface element. As a result, interfacing a metasurface with a two-dimensional electrical addressing architecture would yield a chip-scale optical component capable of performing diverse optical functions.

While the prospect of technological applications motivates a large body of research into active metasurfaces, the question of the fundamental and practical limits of active metasurfaces in handling high laser powers used in many applications is still to be determined (>1 kW/cm^2^ to 10s kW/cm^2^ irradiances in the case of steady-state illumination or peak powers >100 kW/cm^2^ in the case of illumination with short laser pulses). The challenges arise from the nature of the metallic or metal-like constituents in many metasurfaces that are expected to be lossy to the point where a large fraction of the incoming light is converted to potentially damaging heat. A recent paper [39] has experimentally investigated laser damage performance of thin Au, ITO, and TiN films, which can be used as key constituent layers of active nanophotonic structures. However, the laser damage thresholds for these films are expected to significantly decrease when these films are integrated into resonant nanophotonic structures such as metasurfaces due to electric field enhancement in these structures. Therefore, it is important to estimate the peak power that a prototypical actively tunable metasurface can handle without being damaged, for both pulsed and continuous wave (CW) illumination. Despite the fact that ITO-based plasmonic active metasurfaces have demonstrated the ability to actively manipulate the wavefront of reflected light, they have only been tested under low (mW/cm^2^) laser irradiances. Identifying upper power performance limits of our gate-tunable active metasurfaces would set the scope of potential practical applications for such devices.

Here, we theoretically and experimentally investigate the thermal performance of gate-tunable conducting oxide metasurfaces illuminated by high-irradiance laser beams. Based on our previous work, we designed a gate-tunable ITO-based active metasurface [18] which exhibits an electrically tunable optical response. At an optical communication wavelength of 1550 nm, our metasurface exhibits a reflectance of 13.5%, which drops dramatically under applied bias when the charge accumulation layer of ITO undergoes an epsilon-near-zero (ENZ) transition. We then simulate the related thermal response of the designed metasurface both under CW and pulsed laser illumination. We identify the key properties, which determine the temperature excursion in the illuminated metasurface. To validate our calculations, we fabricate an ITO-based active metasurface and experimentally probe its gate-tunable optical response upon CW illumination by high-irradiance laser beams. Additionally, we design an actively tunable metasurface using a less lossy transparent conducting oxide, cadmium oxide (CdO), as an electrically tunable active layer alternative to ITO. With the same geometrical parameters as for ITO-based metasurface, our CdO-based active metasurface exhibits relatively high reflectance (R) at all applied voltages (R > 22%) at an operating wavelength of 1559 nm. CdO-based active metasurface also exhibits a higher laser damage threshold, due to reduced absorbance and a higher thermal conductivity for CdO compared to ITO. Our metasurface designs are found to be relatively robust and potentially suitable for many high-power industrial applications such as beam steering and shaping that could find practical use in laser machining or additive manufacturing. We also show that our metasurfaces can support irradiances necessary for long range free-space optical communication and LiDAR applications, and provide estimates for the communication and ranging distances for our metasurfaces in LiDAR, free-space optical communication systems, and remote gas sensing applications [40].

## 2 Results and discussion

### 2.1 Design and optical performance of ITO-based gate-tunable metasurfaces

**Figure 1:**
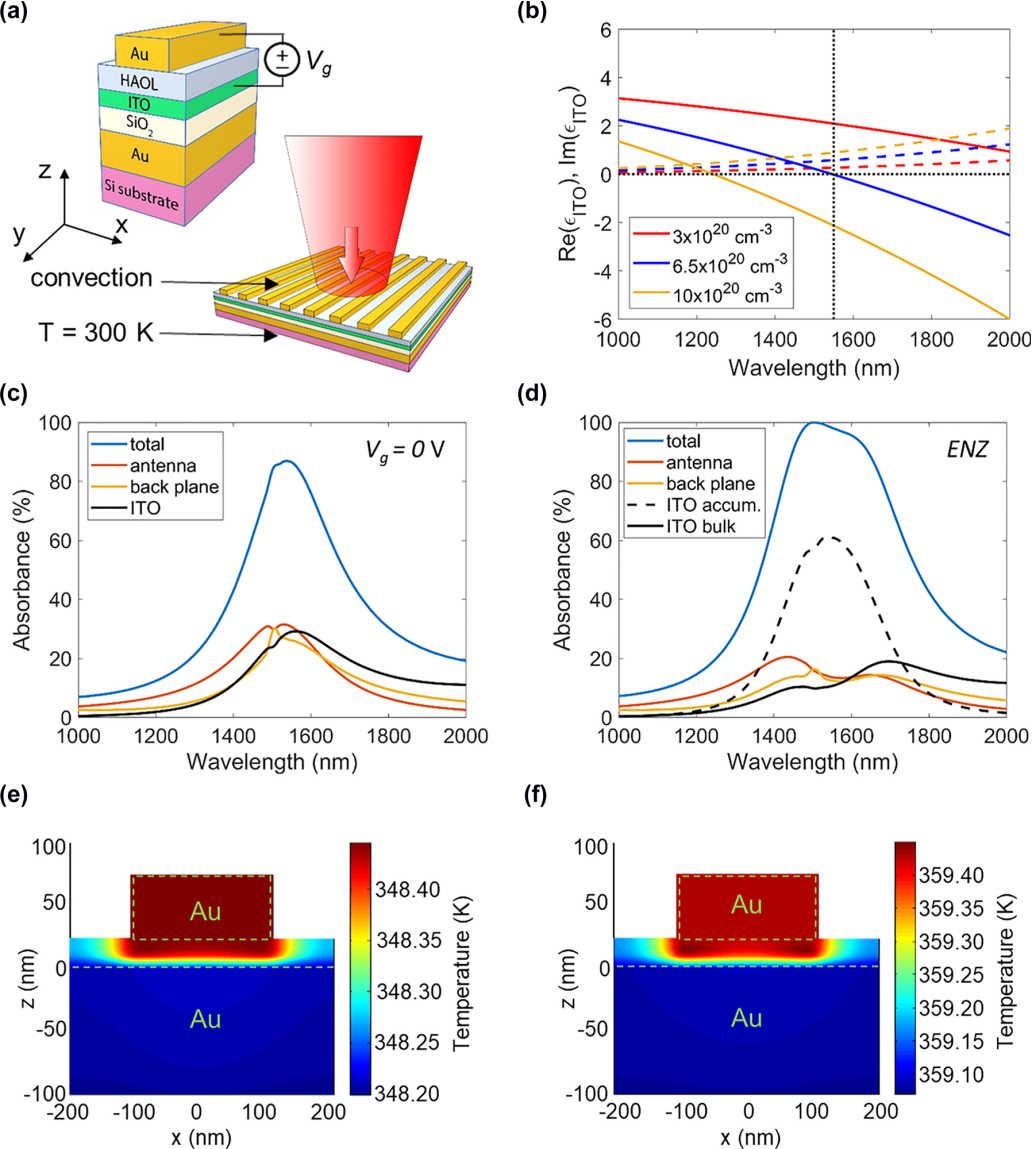
Optical and thermal properties of the ITO-based gate-tunable metasurface. (a) Schematic of the metasurface unit cell. (b) Real (solid lines) and imaginary (dashed lines) parts of the complex dielectric permittivity of ITO *ɛ*_ITO_ as a function of wavelength for different values of the carrier density of ITO. (c) and (d) Absorbance spectra in different material layers of our gate-tunable metasurface in the cases of (c) no applied bias and (d) in the case when the accumulation layer of ITO is in the ENZ regime (*N*_acc_ = 6.5 × 10^20^ cm^−3^). In (c) and (d), the blue lines show absorbance spectra of the metasurface. (e) Spatial distribution of temperature inside the metasurface unit cell in the case of no applied bias. (f) Spatial distribution of temperature inside the metasurface unit cell for the case when the accumulation layer of ITO is in the ENZ condition (*N*_acc_ = 6.5 × 10^20^ cm^−3^). In (e) and (f), we assume CW laser illumination, the thickness of the Si substrate is taken as 250 μm, and the assumed irradiance is 3.5 kW/cm^2^.

Our metasurface motif is based on a plasmonic reflectarray structure [18] shown in Figure 1(a). Each metasurface antenna consists of an 80 nm-thick Au back plane followed by a 10 nm-thick SiO_2_ layer, a 5 nm-thick ITO layer, and a 10 nm-thick layer of HfO_2_/Al_2_O_3_ nanolaminate (HAOL) [19]. On top of the dielectric HAOL layer, we place a 50 nm-thick Au wire, which is much longer in the *y* direction. The chosen wire width of 216 nm ensures that the metasurface supports a magnetic dipole resonance at a wavelength of 1550 nm. Our metasurface features a periodic arrangement of antennas, with a period of 400 nm (see the bottom section of Figure 1(a)). Our full wave simulations show that placing a lower refractive index layer between the Au back plane and the ITO layer enhances the reflectance of the metasurface. Thus, in our metasurface design, there is a 10 nm-thick SiO_2_ layer placed between the Au back reflector and the ITO layer.

When the Au wire and the ITO layer are biased with respect to each other, a thin electron accumulation or depletion layer is formed in the ITO at the interface with the HAOL layer (Supplementary Materials, Part 2). Device physics calculations show that for a bulk ITO electron density of 3 × 10^20^ cm^−3^, the thickness of the ITO accumulation/depletion layer is around 1 nm [18]. We also refer to the charge accumulation/depletion layer in the ITO film as an active ITO layer. Figure 1(b) plots the real and imaginary parts of the dielectric permittivity of ITO as a function of wavelength for a range of values of the ITO carrier density *N.* As seen in Figure 1(b), at a given wavelength, the real part of the dielectric permittivity of ITO Re(*ɛ*_ITO_) decreases with the electron density *N.* On the other hand, the imaginary part of the dielectric permittivity of ITO Im(*ɛ*_ITO_) increases with the electron density *N.* When the carrier density of ITO reaches *N* = 6.5 × 10^20^ cm^−3^, Re(*ɛ*_*ITO*_) = 0 at an operating wavelength of 1550 nm (see Figure 1(b)). When the carrier density of ITO (*N*) is further increased, Re(*ɛ*_ITO_) becomes negative. This transition of Re(*ɛ*_ITO_) from positive to negative values is typically referred to as an ENZ transition. The ENZ transition in semiconductors plays an important role in a number of optoelectronic devices because it marks a transition between dielectric and metallic response [41, 42].

The phase of the light reflected from ITO-based gate-tunable metasurfaces can be significantly modified when the active ITO layer undergoes an ENZ transition under an applied bias [18], [19], [20, 43]. First, we perform full wave electromagnetic simulations to calculate electrically tunable phase and amplitude response of the designed metasurface. In our full wave simulations, we assume that the metasurface antenna is infinite in *y* direction, and we use periodic boundary conditions in *x* direction (Figure 1(a)) to represent a periodic array of antennas. Our calculations show that the ITO metasurface provides a broad phase shift under applied bias across the whole telecommunication C-band, which spans from 1530 nm to 1560 nm (Supplementary Materials, Part 3). At an operating wavelength of 1550 nm, under applied bias (*V*_g_) the accumulation regime (*V*_g_ > 0) contributes up to 300° of the 315° phase shift produced by the metasurface, with the depletion regime (*V*_g_ < 0) contributing 15° to the phase shift balance. At a wavelength of 1550 nm, the calculated reflectance without applied bias is 13.5%. However, under applied electrical bias, when the accumulation layer of ITO is in the ENZ region, the reflectance can be as low as 2.7%. Previously reported ITO-based gate-tunable plasmonic metasurfaces have also exhibited quite modest reflectance values [18–20]. It is not clear; however, which material layers in the metasurface structure cause this high absorbance.

Therefore, to better understand which material layers contribute most to the metasurface absorbance, we calculate absorbance in each of the constituent layers (Figure 1(c) and (d)). The absorbance in a given layer has been defined as the fraction of the incoming light energy which is absorbed by that layer. As seen in Figure 1(c), when no external bias voltage is applied, the Au back plane, the top Au wire, and the ITO layer absorb almost equal amounts of incoming light at resonant wavelengths (around 30%). However, under applied bias, when the ITO accumulation layer is in the ENZ regime, the absorbance in the ITO layer exceeds 70%. This dramatic absorbance increase in the ITO layer is accompanied by a significant absorbance decrease in the Au layers from a peak of about 30% to less than 20%. Since our 5 nm-thick ITO layer is divided into a 4 nm–thick non-modulated ITO and a 1 nm-thick accumulation layer, we calculate absorbance in each of these ITO regions (Figure 1(d)). Our calculations show that in the ENZ regime, the 4 nm–thick non-modulated ITO layer absorbs less than 10% of the incoming light. On the other hand, in the ENZ regime, absorbance in the 1 nm–thick ITO accumulation layer is above 60% for a broad range of resonant wavelengths (Figure 1(d)). Since in the ENZ regime a large fraction of the incoming light is absorbed in the 1 nm–thick active ITO layer, it was not clear *a priori* how the metasurface will respond thermally when exposed to a high-power laser beam. Will this localized optical absorption result in heating of our metasurface above damage threshold when applying electrical bias?

### 2.2 Analysis of thermal response of ITO-based gate-tunable metasurfaces: CW illumination

To answer the above question, we calculate the temperature increase inside the metasurface by employing a routinely used simulation protocol [44]. First, we use full wave simulations to calculate the spatial distribution of the electric field inside the metasurface. We use the derived electric field distribution to calculate the power absorbed per unit volume (absorption density) *h*(*r*) according to $h\left(r\right)=\frac{1}{2}\omega \varepsilon_{0}Im\left(\varepsilon \right)|E\left(r\right){|}^{2}$ . Here, *ω* is the angular frequency of the incoming laser light, *ɛ* is the complex dielectric permittivity of the medium, and *E*(*r*) represents the complex amplitude of the optical electric field. Next, we use the calculated absorbed power density as an input to numerically calculate the spatial temperature distribution inside the metasurface using a commercial software package [45]. In our thermal simulations, we calculate a projected temperature increase in an infinite planar metasurface (see the bottom panel of Figure 1(a)). Thus, our simulation approach does not account for heat conduction in lateral directions. We also assume that the temperature at the bottom of the substrate is heat-sinked at a fixed temperature of 300 K, while the temperature of the metasurface and other parts of the substrate are unconstrained (see Figure 1(a)).

In practice, this boundary condition can be realized, e.g., by flowing coolant liquid at the bottom of the substrate. This thermal management configuration, which relies on the coolant flow, is routinely used for heat management in optical components such as laser diodes or thin disk gain media. Note that the total thickness of the metasurface layer (including the Au back plane) is less than 200 nm while the considered substrate thickness is at least tens of microns. Ultrathin nature of metasurfaces implies that upon high-power illumination the localized temperature increase in metasurfaces may be quite high due localized absorption [46]. However, the localized nature of temperature increase also facilitates heat withdrawal from the metasurfaces when the metasurface is heat-sinked. Using the described framework, we can calculate the temperature distribution inside the metasurface assuming the spatial shape of the laser light is that of a uniform plane wave. When analyzing ITO-based, gate-tunable metasurfaces, we assume an illumination wavelength of 1550 nm. We fix the irradiance and observe the general trends of the spatial distribution of temperature inside the metasurface. We take the irradiance of the incoming laser light to be 3.5 kW/cm^2^. Note that we define the irradiance as the power of the laser light incident on a unit area of the metasurface. In our heat simulations, the metasurface is built on a 250 μm-thick Si substrate. The modeled infinite planar metasurface is able to withstand irradiances as high as 3.5 kW/cm^2^ due to the presence of the heat sink implicitly incorporated in our model by fixing the temperature to *T* = 300 K at the bottom of the substrate. We observe a steady state temperature increase of around 50 K above the ambient of 300 K while the temperature variation inside the metasurface (from the back reflector to a top Au wire) is less than 0.3 K (see Figure 1(e)), which would limit interlayer mechanical stresses due to thermal expansion.

Next, we examine how the thermal response of the metasurface varies under bias, and the real part of the dielectric permittivity of the active ITO layer Re(*ɛ*_ITO_) is in the ENZ region. Under bias, we assume that the carrier density of the 1-nm–thick active ITO layer is *N*_acc_ = 6.5 × 10^20^ cm^−3^ (Figure 1(b)). In this active switching case, we indeed observe that the highest temperature is achieved in the ITO accumulation layer (Figure 1(f)). However, the temperature increase in the accumulation layer of ITO, as compared with the temperatures of the surrounding layers, is limited despite a significant electric field enhancement in the active ITO layer. In other words, the ultrathin ITO accumulation layer can effectively dissipate heat even when driven to high absorbance levels. In contrast, in the case of zero applied bias, the highest temperature is attained in the Au wire (Figure 1(e)).

### 2.3 Thermal performance of ITO-based gate-tunable metasurfaces: pulsed illumination

**Figure 2:**
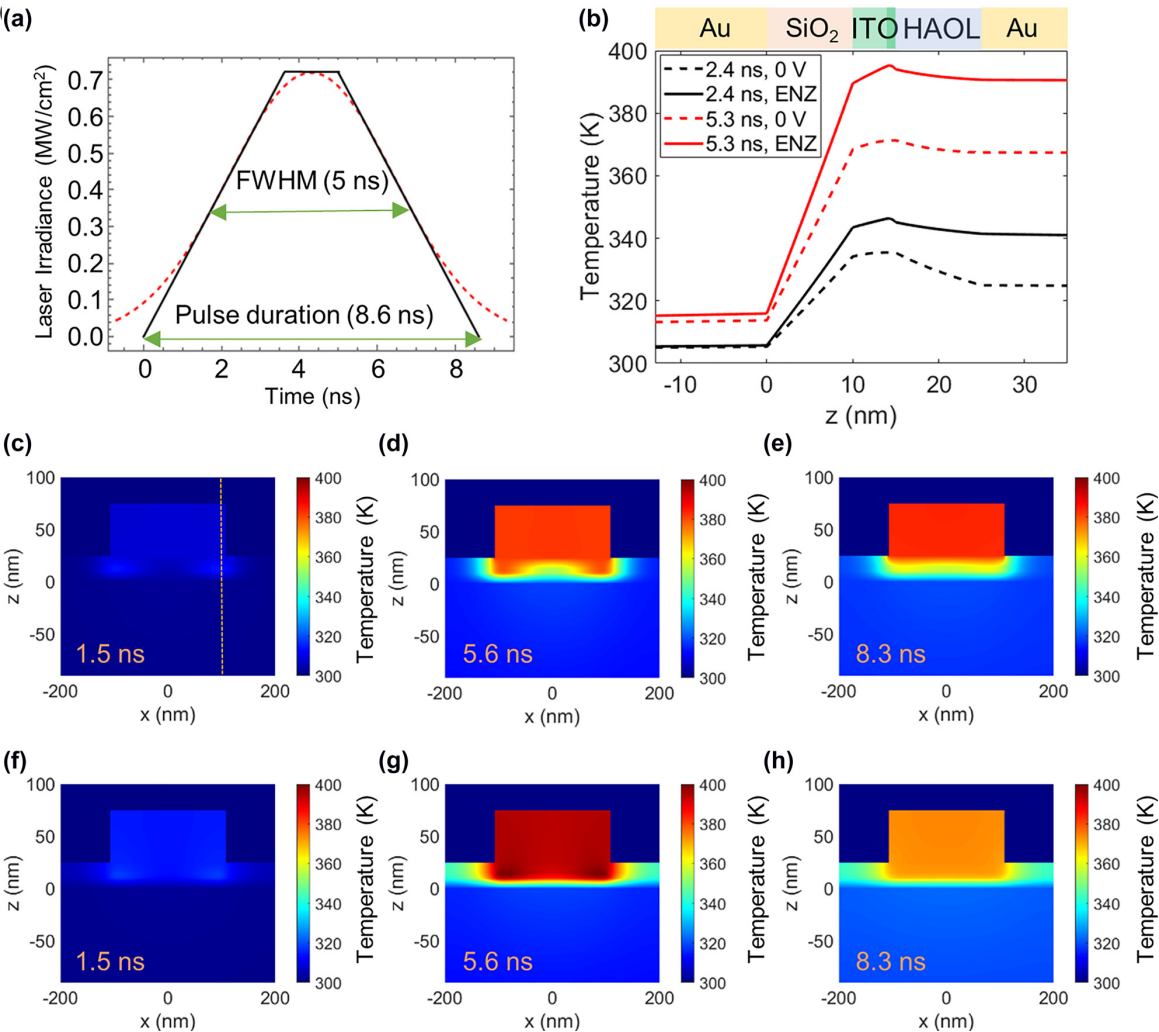
Thermal response of the ITO-based gate-tunable metasurface from exposure to pulsed laser illumination. (a) Laser irradiance as a function of time. In our simulations, we approximate a Gaussian pulse (red dashed line) with a trapezoidal pulse (black solid line). (b) Temperature inside the metasurface element as a function of *z* coordinates at *x* = 100 nm (dashed line in (c)). Red and black curves correspond to the temperature distributions during the pulse 2.4 ns and 5.3 ns after turning on the laser pulse, respectively. Dashed lines correspond to the case of no applied bias while solid lines correspond to the case when the ITO accumulation layer is in the ENZ regime (*N*_acc_ = 6.5 × 10^20^ cm^−3^). (c)–(e) Spatial distribution of temperature inside the metasurface in the case of no applied bias 1.5 ns, 5.6 ns, and 8.3 ns after turning on the laser pulse, respectively. (f)–(h) Spatial distribution of temperature inside the metasurface in the case when the ITO accumulation layer is in the ENZ regime (*N*_acc_ = 6.5 × 10^20^ cm^−3^) at 1.5 ns, 5.6 ns, and 8.3 ns after turning on the laser pulse, respectively.

Next, we theoretically investigate transient thermal behavior of the metasurface when it is illuminated by a single 5 ns-long laser pulse. The spatial distribution of temperature inside the metasurface can be quite different than the case of the steady-state CW illumination. In practical situations, the temporal shape of the laser profile is typically Gaussian. Here, we model the situation where the full width at half maximum (FWHM) of the considered laser pulse is *τ*_p_ = 5 ns, corresponding to laser sources typically employed in LiDARs. Since the software package we use does not allow the implementation of Gaussian-shaped laser pulses, we approximate the pulse with a trapezoidal one (Figure 2(a)), which approximates very closely the energy in the Gaussian pulse and its peak power. The laser fluence of the considered pulse is 3.5 mJ/cm^2^ (corresponding to a peak laser irradiance of 0.7 MW/cm^2^), and the total duration of the considered trapezoidal pulse is 8.6 ns.

Figure 2(b) plots the spatial temperature distribution inside the metasurface as a function of *z* coordinate when the *x* coordinate is fixed at *x* = 100 nm (see the dashed vertical line in Figure 2(c)). The plotted temperature curves correspond to two different fixed values of time, 2.4 ns and 5.3 ns after the laser pulse has been turned on. The solid curves correspond to the case of no applied bias while the dashed curves correspond to the case where the active ITO layer is in the ENZ regime (*N*_acc_ = 6.5 × 10^20^ cm^−3^). The plane *z* = 0 corresponds to the interface of the back reflector and the SiO_2_ layer (Figure 1(a)). As expected, we observe that applying electrical bias results in a higher peak temperature in the metasurface. We also observe a significant temperature difference at two different interfaces of the 10 nm-thick SiO_2_ layer. This significant temperature difference is caused by the low thermal conductivity of the amorphous SiO_2_ film. Thus, we observe that for the considered laser fluence of 3.5 mJ/cm^2^, the temperature difference between the back plane and the top Au wire can be as high as 70 K, potentially resulting in the build-up of the inter-layer mechanical stress. For the peak laser irradiance of 0.7 MW/cm^2^ the peak temperature of the metasurface is slightly below 400 K, where we expect reasonable electronic performance of our metasurface device [47]. As seen in Figure 2(b), ITO is the hottest layer of the metasurface since it is sandwiched between two amorphous insulating dielectric layers, SiO_2_ and HAOL, with low thermal conductivities. We also observe that in the case when the ITO accumulation layer is in the ENZ regime, the highest temperature is attained in the 1 nm–thick active ITO layer. Thus, unlike the case of the CW laser illumination, pulsed laser illumination results in significant temperature variation in the metasurface because on nanosecond time scales, the thermal diffusion length is limited and most of the absorbed power raises the temperature rather than diffusing out of the metasurface heterostructure as discussed below.

When the pulse duration is on the order of nanoseconds, the temperature of the metasurface does not depend on substrate thickness. This insensitivity towards the substrate thickness is expected, since the thermal diffusion length in the Si substrate, *μ*_*t*_, is much smaller than the thickness of the substrate (100 µm). Indeed, taking into account the thermal diffusivity of Si (*D* = 8.9 × 10^−5^ m^2^/s), we calculate the thermal diffusion length in the Si substrate, *μ*_*t*_, according to $\mu_{t}=2\sqrt{D\tau_{\text{p}}}$ , where *τ*_p_ = 5 ns corresponds to the FWHM of the laser pulse (Figure 2(a)). The estimated thermal diffusion length is *μ*_*t*_ = 1.3 μm, which is indeed much smaller than the substrate thickness. Our simulations also show that ∼140 ns after the laser pulse has been turned off, the temperature of the metasurface is reduced by a factor of e^3^ (Supplementary Materials, Part 7). This indicates that the repetition rate of the pulsed laser used for illuminating metasurface can be ∼6.7 MHz. On the other hand, in the pulsed illumination regime, the dynamic temperature rise and the peak metasurface temperature will be considerably affected by thermal conductivities of the constituent layers’ thicknesses nearby highly absorbing regions. For example, replacing a low thermal conductivity SiO_2_ layer with a layer of higher thermal conductivity material will reduce the peak temperature attained in the metasurface. In contrast, in the CW illumination regime, changing thermal conductivity values of thin films forming the metasurface, e.g., by a factor of two, practically does not affect the temperature of the metasurface. Hence, for a given absorbance, thermal management of metasurfaces in the cases of steady-state and pulsed laser illuminations requires different strategies.

We explore in further detail how the temperature distribution inside the metasurface changes with time in the case of pulsed laser illumination. Unlike the case of the CW illumination regime, for pulsed laser illumination, the local temperature variation within the ultrathin metasurface layer can be quite significant (Figure 2). Figure 2(c)–(h) plot the spatial distribution of temperature in the *x–y* cross-section of the metasurface (see Figure 1(a)) 1.5 ns, 5.6 ns, and 8.3 ns after the laser pulse has been turned on. Figure 2(c)–(e) correspond to the case of no applied bias while Figure 2(f)–(h) correspond to the case when the active ITO layer is in the ENZ regime (*N*_acc_ = 6.5 × 10^20^ cm^−3^). As seen in Figure 2(c), as the metasurface starts interacting with the laser pulse, we attain the highest temperature in the dielectric heterostructure (SiO_2_/ITO/HAOL), more specifically, the temperature is highest in the ITO layer. Moreover, there is a significant temperature variation under the Au wire in the *x* direction. Later on, 5.6 ns and 8.3 ns after the laser pulse has been turned on, the highest temperature in the metasurface is attained in the top Au wire rather than in the dielectric heterostructure (Figure 2(d)–(e)). We also observe that closer to the end of the laser pulse (8.3 ns) the temperature of the dielectric heterostructure (under the Au wire) does not vary laterally (Figure 2(e)). The trends observed in the case of applied bias (Figure 2(f)–(h)) are overall similar to the trends observed in the case of no applied bias (Figure 2(c)–(e)). We observe that in the case of applied bias, the peak temperature is attained at somewhat different points in time compared to the case of no applied voltage. Figure 2(g) shows that 5.6 ns after the laser pulse has been turned on, the highest temperature is still observed in the ITO accumulation layer. We also observe that 5.6 ns and 8.3 ns after the laser pulse has been turned on, the temperature of the metasurface under applied bias is higher than the temperature of the metasurface without applied bias. Thus, in the case of the transient laser illumination, the temperature variation under the Au wire can be significant both in the vertical *y* and lateral *x* directions.

**Figure 3:**
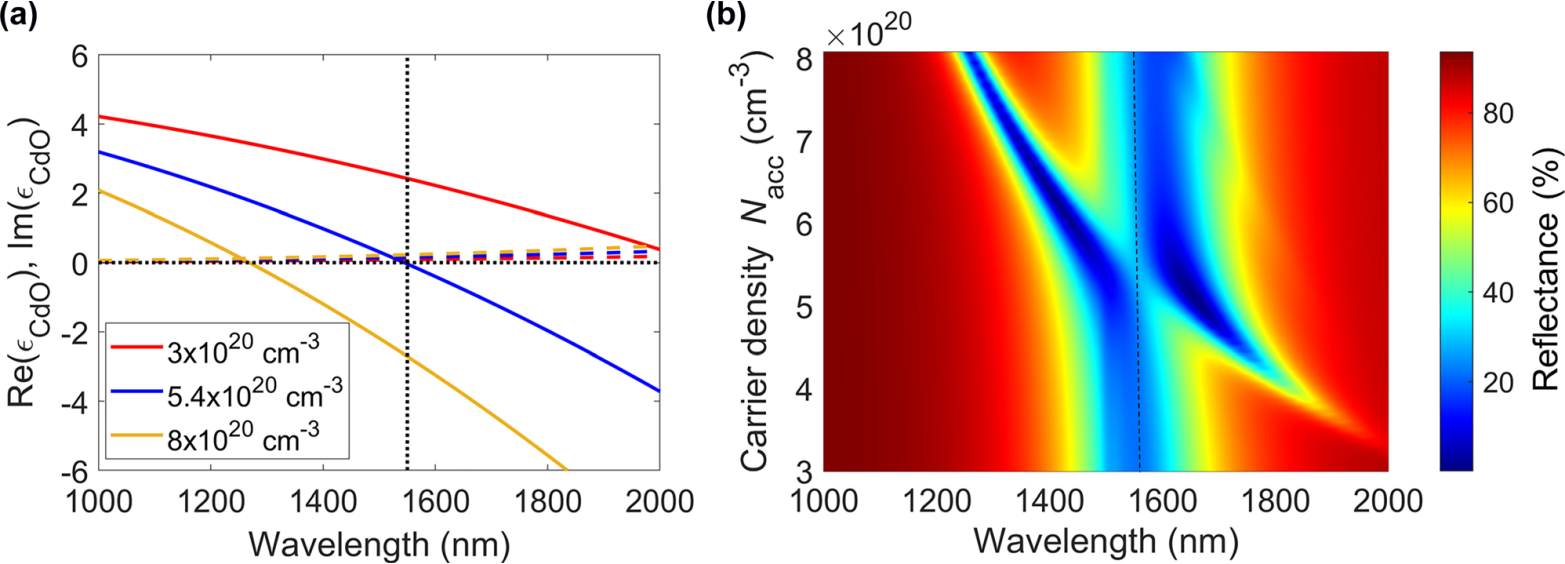
Optical performance of CdO-based gate-tunable metasurfaces. (a) Real (solid lines) and imaginary (dashed lines) parts of the complex dielectric permittivity of CdO, *ɛ*_CdO_, as a function of wavelength for different values of the carrier density of CdO. (b) Reflectance of the CdO-based gate-tunable metasurface as a function of wavelength and carrier density in the CdO accumulation layer.

Consider a hypothetical metasurface with dimensions of 0.2 × 0.2 cm^2^, which is illuminated by a laser beam of *D*_beam_ = 0.2 cm in diameter. To understand the potential of such mid-size metasurfaces to contribute to future practical applications, we estimate the detection range of the metasurface-based LiDAR systems and perform link budget analysis for the case of metasurface-based free space optical communications systems (Supplementary Materials, Part 1). For a beam diameter of *D*_beam_ = 0.2 cm, the energy of the laser pulse depicted in Figure 2(a) is 0.11 mJ. For this pulse energy, the detection range of a metasurface-based LiDAR system can approach 1 km. Further, for a given temperature increase, in the metasurface, the pulsed laser irradiation enables larger detection range as compared with CW illumination (Supplementary Materials, Part 1).

### 2.4 CdO-based gate-tunable metasurfaces: optical and thermal performance

Designing gate-tunable metasurfaces which exhibit higher reflectance and lower absorbance could significantly enhance their laser damage threshold. A possible route towards enhancing the metasurface reflectance would be replacing the ITO layer with a less lossy transparent conducting oxide. To illustrate this approach, we consider here the use of cadmium oxide (CdO) active layer instead of ITO. As seen in Figure 3(a), when changing the carrier density, the real part of the dielectric permittivity of CdO Re(*ɛ*_CdO_) undergoes the ENZ transition in the near-infrared wavelength range. Interestingly, the imaginary part of the dielectric permittivity of CdO Im(*ɛ*_CdO_) stays relatively modest. Note that the real part of the dielectric permittivity of CdO Re(*ɛ*_CdO_) exhibits a larger decrease when the carrier density is increased as compared to the real part of the dielectric permittivity of ITO Re(*ɛ*_ITO_) (*cf.* Figures 1(b) and 3(a)). This stronger variability is because the effective electron mass of CdO is lower than that of ITO. This stronger variability also implies that the ENZ regime in CdO can be achieved at lower bias voltages than in the case of ITO. We thus highlight CdO as a good candidate for an improved active layer in our gate-tunable metasurfaces.

To investigate how using CdO could potentially improve the performance of plasmonic metasurfaces, we used the same metasurface element we developed for an ITO-based metasurface (Figure 1(a)) but replaced the 5 nm-thick ITO layer with a 5 nm-thick CdO layer with a carrier density of 3 × 10^20^ cm^−3^. Figure 3(b) plots the reflectance of the CdO-based gate-tunable metasurface as a function of wavelength and carrier density in the 1 nm-thick accumulation layer. As seen in Figure 3(b), even without modifying the geometrical parameters of the metasurface element, the CdO-based metasurface still exhibits the resonance dip around the wavelength of 1550 nm. At no applied bias we observe a resonance dip with a minimal reflectance around 22%. When increasing the carrier density in the accumulation layer, we observe a second reflectance dip at longer wavelengths, which blue shifts when increasing the carrier density in the accumulation layer. This second reflectance dip is associated with increased absorption in CdO at ENZ wavelengths. At carrier densities where the geometrical resonance and the ENZ resonance overlap, the reflectance exhibits an avoided crossing. As seen in Figure 3(b), we can identify a wavelength range for which the reflectance of the metasurface increases with applied bias. For example, at a wavelength of 1559 nm, we observe that the reflectance increases from 22% to 29% when the active CdO layer is in the ENZ regime while exhibiting a large phase shift of 293°. This behavior differs significantly from the behavior observed in case of ITO-based metasurfaces for which the reflectance drops significantly when the active ITO layer is in the ENZ regime. Finally, we would like to highlight that in the proposed design, the toxic CdO layer is encapsulated in the top gate dielectric layer thus limiting the probability of cadmium exposure while handling the metasurface.

**Figure 4:**
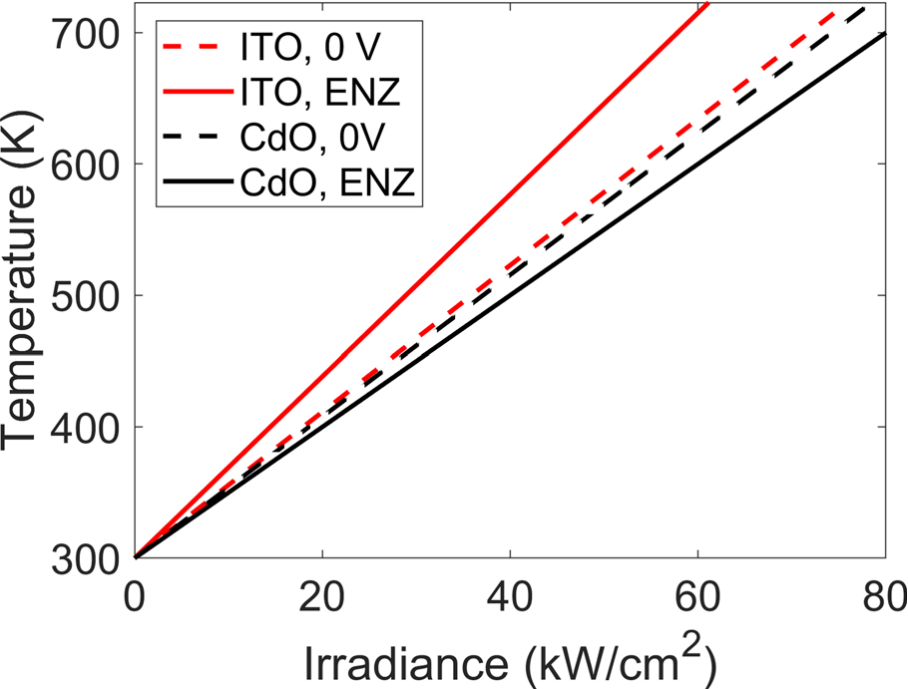
Calculated peak temperature inside the CdO-based and ITO-based gate-tunable metasurfaces as a function of the laser irradiance in the case of CW illumination. The solid lines correspond to the case of zero applied bias while the dashed lines correspond to when the active transparent conductive oxide (CdO or ITO) layer is in the ENZ regime. In case of the CdO- and ITO-based active metasurfaces, the ENZ regime corresponds to carrier densities of *N*_acc_ = 5.4 × 10^20^ cm^−3^ and *N*_acc_ = 6.5 × 10^20^ cm^−3^, respectively. The assumed substrate thickness is 100 μm.

Next, we investigate the thermal performance of the CdO-based gate-tunable metasurface in the CW illumination regime (the case of pulsed illumination is discussed in Supplementary Materials, Part 11). In our simulations, we adopted thermal properties of CdO films reported in the literature [48, 49]. The substrate thickness is taken as 100 μm. When discussing thermal performance of CdO-based metasurfaces we assume an illumination wavelength of 1559 nm. Figure 4 plots the peak temperature in the metasurface as a function of the laser irradiance. As seen in Figure 4, due to lower absorbance, the CdO-based metasurface reaches lower peak temperatures than the case of ITO-based active metasurfaces under the same illumination conditions. Unlike ITO, CdO-based metasurfaces’ peak temperature decreases when the CdO accumulation layer is in the ENZ regime. This reduction of temperature is due to the increased reflectance under applied bias. (The reflectance increases from 22% to 29%). As seen in Figure 4, when no electrical bias is applied to the CdO-based metasurface, the melting temperature of thin Au films (∼723 K [50]) is attained at an irradiance of 80 kW/cm^2^. On the other hand, when the ITO-based active metasurface is in the ENZ regime, the maximal temperature of ∼723 K is attained at an irradiance of 61 kW/cm^2^. To summarize, the improved optical performance of the CdO-based active metasurfaces yields an improved thermal response as well.

Based on the performed thermal analysis, we conclude that CdO-based active metasurfaces are suitable for LiDAR and free space optical communications applications (Supplementary Materials, Part 11). Within the context of future applications, we consider a hypothetical mid-size metasurface with dimensions of 0.2 × 0.2 cm^2^. In our estimates, we have assumed that the diameter of the incoming laser beam is *D*_beam_ = 0.2 cm. In the CW regime, the range of the CdO metasurface-based LiDAR system reaches almost 400 m while the range of the metasurface-based free-space optical communications system approaches 500 km. In case of the pulsed illumination, the LiDAR range may reach 1 km while the range of the free-space optical communication system can approach the mark of 10,000 km.

### 2.5 ITO-based gate-tunable metasurfaces upon high-irradiance CW illumination: experimental demonstration

To experimentally validate the simulations performed for infinitely large metasurfaces (see Figure 1(e) and (f)), one would need to utilize specialized kW-class laser facilities and illuminate thermally packaged large-area gate-tunable metasurfaces with ∼1 kW power laser beams. All these steps pose logistic and engineering challenges and are beyond the scope of the present study. However, we can still gain interesting insight by experimentally investigating gate-tunable performance of an active metasurface illuminated by high-brightness laser sources, albeit with a small footprint. This would enable us to experimentally observe whether the localized heating by the laser source alters the gate-tunable optical response of our active metasurface. As detailed below, in our experiment, we focus the laser beam onto the metasurface down to the spot of 7 μm in diameter and experimentally study its gate-tunable performance for different values of irradiances. The laser spot of 7 μm in diameter illuminates around 17 metasurface unit cells, and, hence, this approach still provides an insight regarding metasurface performance rather than probes the optical response of a single scatterer. We also use distinct modelling approach to estimate the temperature increase for the case when the focused laser beam illuminates the metasurface (see Supplementary Materials, Part 7)

**Figure 5:**
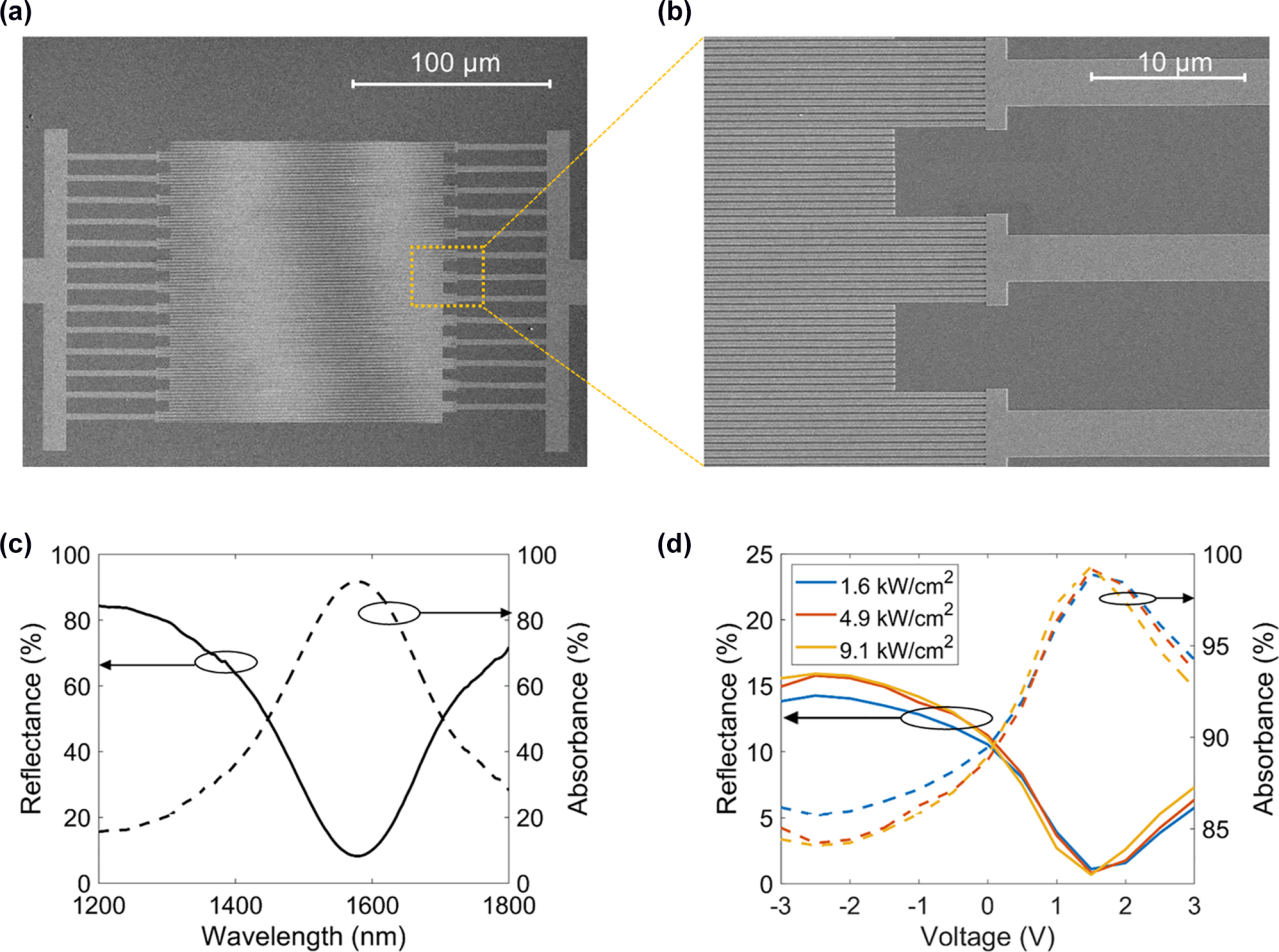
Metasurfaces upon high-irradiance CW illumination: experimental results. (a) and (b) SEM images of the fabricated ITO-based gate-tunable metasurface. (c) Measured reflectance spectrum (solid line) and absorbance spectrum (dashed line). (d) Experimental reflectance (solid lines) and absorbance (dashed lines) as a function of voltage for different irradiances of the incoming laser beam. In (c) and (d), the absorbance was inferred from the measured reflectance. The laser beam illuminating the metasurface was 7 µm in diameter. The wavelength was fixed to λ = 1555 nm.

The scanning electron microscopy (SEM) images of the fabricated reflectarray metasurface are shown in Figure 5(a) and (b). The central part of Figure 5(a) shows the stripe antenna array while the Au electrodes are visible in the left and right sides of the image. Figure 5(b) shows the enhanced image of the metasurface edge. The fabricated sample is comprised of a Si substrate, a 1 μm-thick layer of SiO_2_, an Au back reflector, a 17 nm-thick ITO layer, an 8 nm-thick Al_2_O_3_ gate dielectric, and Au stripe-shaped patch antennas (see the inset of Figure S9). For fabrication simplicity, we omitted the thin SiO_2_ layer between the back reflector and ITO, which is seen in our original design (Figure 1(a)). The thickness of the Au patch antenna is 40 nm, and its width is 260 nm. As a first step, we measured the reflectance spectrum upon low-power illumination (Figure 5(c)). As expected, we observe a broad plasmonic resonance in the near-infrared wavelength range. Next, we focused the CW laser beam onto the metasurface so that the diameter of the laser spot was 7 μm and measured the reflectance as a function of applied voltage (Figure 5(d)). In our measurement, we fixed the operating wavelength at *λ* = 1555 nm (at the resonance) and considered three different irradiances, 1.6 kW/cm^2^, 4.9 kW/cm^2^, and 9.1 kW/cm^2^. The highest laser power used in our experiment is 3.5 mW. A recent work has studied the thermal behavior of thin films upon focused illumination by a 1 mW laser beam [46]. As reported in the mentioned reference [46], in the wavelength range where absorbance is high, the temperature increase in thin metal films is non-negligible. Hence, it is not evident *a priori* whether the metasurface would exhibit an adequate gate-tunable optical response at the illuminating power of 3.5 mW, considering that under applied bias at the wavelength of interest the metasurface absorbance can be as high as 99.3% and that the ITO layer exhibits extreme localized absorbance in the ENZ regime.

In our measurements, we measure the metasurface reflectance at a wavelength of *λ* = 1555 nm while gradually increasing the applied voltage from −3 V to 3 V, where the electron density in the ITO layer at the ITO/Al_2_O_3_ interface is increased, and the ITO accumulation layer is in the ENZ regime (see Figure 5(d)). At each voltage step, we record the reflectance at three different irradiance values: 1.6 kW/cm^2^, 4.9 kW/cm^2^, and 9.1 kW/cm^2^. We observe that the absolute variation of the reflectance at a given irradiance is 15%. Importantly, the electrically tunable optical response of the metasurface is practically unaffected by increased laser powers (Figure 5(d)).

To estimate the temperature of the metasurface upon focused laser illumination, we model the metasurface as a heated disc of diameter *d* on a semi-infinite substrate such that the boundary condition *T* = 300 K is at locations well removed from the heated disc (Supplementary Materials, Part 7) [51]. Prior research has shown that this methodology can accurately describe experimental results [52]. This simple model projects that at an irradiance of 9.1 kW/cm^2^, absorbance of 99.3%, and for a laser spot diameter of 7 μm, a temperature increase of the metasurface an SiO_2_ substrate is 167 K. In principle, the increased temperature could affect the optical properties of thin material layers (Au, Al_2_O_3_, ITO), which constitute the fabricated metasurface.

To understand how the increased temperature may affect the resonant optical response of the metasurface, we measure the complex dielectric permittivities of resistively heated thin Au, Al_2_O_3_, and ITO films at temperatures up to 423 K. (Supplementary Materials, Part 8). We observe that the optical constants of an ITO film encapsulated by an 8 nm-thick Al_2_O_3_ film are unchanged at temperatures up to 423 K. Note that in our metasurface designs the ITO film is encapsulated by either Al_2_O_3_ or HAOL films contributing to the robustness of the optical response of the metasurface at elevated temperatures. Additionally, we performed high-irradiance cycling at three distinct spots of the metasurface and performed dynamically switchable diffraction measurements after the cycling has been completed (Supplementary Materials, Part 14). We observed that the high-irradiance cycling experiments did not alter the far-field diffraction pattern generated by the metasurface in a noticeable way.

## 3 Conclusions

In summary, we have investigated the thermal performance of gate-tunable conducting oxide metasurfaces which are illuminated with high-power laser beams under both CW and pulsed laser illumination conditions. Our gate-tunable metasurfaces use ITO or CdO as active light-modulating materials, which undergo an ENZ transition under applied electrical bias. As compared with ITO-based active metasurfaces, CdO-based active metasurfaces yield a relative reflectance increase of 63% at zero applied bias and a reflectance increase by a factor of 10 when the active layer is in the ENZ regime. We have shown that even under an applied bias, where over 60% of the incoming light is absorbed in a 1 nm–thick active ITO (or CdO) layer, the localized optical absorption does not necessarily result in localized heating of our metasurface above the melting damage threshold criterion used in this study. We have experimentally probed the gate-tunable performance of ITO-based active metasurfaces upon CW high-irradiance illumination while focusing the laser beam onto the metasurface so that the laser spot diameter was 7 μm. We observed that at irradiances 9.1 kW/cm^2^ and 4.9 kW/cm^2^, the electrically tunable optical response of the metasurface was similar to that for lower irradiance of 1.6 kW/cm^2^. Designing active metasurfaces with higher reflectance would further increase the laser powers that these metasurfaces can support. Based on our thermal analysis, we conclude that our metasurfaces can support irradiances necessary for LiDAR, gas sensing, and long-range optical communication applications and may even be considered in industrial applications involving laser-based manufacturing.

## 4 Methods

### 4.1 Full wave optical simulations of active metasurfaces

The optical response of our metasurfaces was calculated using the finite difference time domain method (FDTD Lumerical). In our optical simulations, a normally incident plane wave is illuminating the metasurface from the top (Figure 1(a)). We use the perfectly matched layers (PML) boundary condition in the *z* direction and the periodic boundary conditions in the *x* and *y* directions. Thus, we model the case when the top Au wire is infinite in the *y* direction. Optical response of the metasurface under an applied electrical bias was modeled by describing the charge accumulation/depletion layer in ITO as a 1 nm-thick layer in which the electrical charge is distributed homogeneously. Thus, in our simulations we assumed that in the 1 nm-thick accumulation layer, the carrier density does not vary in the *z* direction. Prior research has also shown that this approximation is capable of capturing the essential features of the optical response of active metasurfaces [53]. We used Gauss’s law to estimate the carrier density increase in this 1 nm-thick accumulation/depletion layer when the ITO layer is biased with respect to the Au back plane (Supplementary Materials, Part 2). The phase the plane wave acquired due to the interaction with the metasurface as well as the reflectance were calculated by extracting the complex electric field from the point monitor placed above the plane wave source.

The absorbance in each of the constituent metasurface layers was calculated by using two different methods. The first method relies on the FDTD simulation. In our FDTD simulation, we place two two-dimensional power monitors, which encompass the considered material layer, and calculate the transmission difference between the two two-dimensional monitors. These two two-dimensional monitors, which are perpendicular to the *z* direction, span the whole simulation area in the *xy* plane. The calculated transmission difference corresponds to the absorbance in the considered layer since the two two-dimensional monitors encompass the mentioned layer. The second method to calculate the absorbance in the constituent layers of the metasurface relies on the discontinuous Galerkin time domain (DGTD) method. Using DGTD simulations (DGTD Lumerical), we extract the absorption density in the metasurface, which then can be used to calculate the absorbance in each of the metasurface layers (Au wire, ITO, Au back plane). The absorption density (absorbance per unit volume) is calculated according to $h\left(r\right)=\frac{1}{2}\omega \varepsilon_{0}Im\left(\varepsilon \right)|E\left(r\right){|}^{2}$ . Absorbance calculated via DGTD and FDTD methods yielded identical results.

### 4.2 Thermal simulation approach

Numerical thermal dynamics of metasurfaces under plane wave or pulsed laser illumination was performed using finite element method (Heat Lumerical). Our thermal simulations are coupled with optical DGTD simulations. As a first step, we calculate the spatial distribution of the absorption density in the metasurface via optical DGTD simulations. Next, the calculated absorption density is imported into the thermal simulation software and the scaling factor which defines the irradiance value is specified. In the thermal simulations, we assume that the temperature of the ambient is 300 K, and the temperature at the bottom of the substrate is also fixed to 300 K (see Figure 1(a)). We also assumed that the top of the metasurface is cooled convectively via natural convection with a convective heat transfer coefficient of 10 W/(m^2^K). When assuming the Si substrate thickness of 250 μm and the irradiance of 3.5 kW/cm^2^, the power flux through the fixed-temperature boundary equals to 27 W/cm^2^, which is orders of magnitude lower as compared with the highest state-of-the-art heat flux values of 1.1 kW/cm^2^ [54]. The materials parameters used in our simulations are summarized in Supplementary Materials, Part 12.

### 4.3 Fabrication of active metasurfaces

We fabricate our metasurfaces on Si substrates with a 1 μm-thick, thermally grown SiO_2_ layer on top. First, we use photolithography to pattern the Au back reflector and back electrode. After developing the exposed photoresist, we use electron-beam (e-beam) evaporation to deposit a 5 nm-thick titanium (Ti) adhesion layer followed by an 80 nm-thick gold (Au) layer. Then, we remove the resist and excess Ti/Au film via a lift-off process in Remover PG. Next, we use photolithography to pattern the ITO layer. Once the exposed resist is developed, we use room-temperature RF magnetron sputtering to sputter a 17 nm-thick ITO film. The process pressure is 3 mTorr, and the applied RF power is 48 W. In the sputterring system, we strike the plasma by using argon (Ar) gas with a flow rate of 20 sccm, and argon/oxygen gas (Ar/O_2_:90/10) with a flow rate of 0.6 sccm. We can change the flow rate of the argon/oxygen gas to control the carrier concentration of the deposited ITO layer and used a flow rate of 0.6 sccm for the device shown in this work. The resist and excess ITO are lifted off in Remover PG. In the next step, we use atomic layer deposition (ALD) to deposit an 8 nm-thick Al_2_O_3_ layer through a shadow mask. Then, we spin a bilayer e-beam resist and use e-beam lithography (EBL) [Raith EBPG 5000+] to pattern Au nanoantennas and top electrodes. During EBL, we use an acceleration voltage of 100 keV and a write dose of 1000 μC/cm^2^. The exposed e-beam resist layer is then developed in a mixture of isopropyl alcohol (IPA) and methyl isobutyl ketone (MIBK), and we use e-beam evaporation to deposit 2 nm of Ti followed by 40 nm of Au. After lifting off the e-beam resist and excess films in Remover PG, we use photolithography to pattern the top and back contact pads followed by e-beam evaporation to deposit a Ti/Au film (20 nm/200 nm). After lift-off in Remover PG, the contact pads are wire-bonded from the metasurface sample to conducting pads on a sample mounting printed circuit board (PCB). The sample mounting PCB is controlled via a voltage-driving PCB.

## Supplementary Materials

The Supplementary Materials are available free of charge at: https://doi.org/10.1515/nanoph-2022-0594.

Link budget analysis and requirements for additive manufacturing; Electrical and optical properties of ITO and CdO; Phase shift and reflectance modulation by ITO-based active metasurfaces; Absorbance in ITO-based active metasurfaces; Spatial distribution of the electric field inside ITO-based active metasurfaces; Thermal behavior of ITO-based active metasurfaces: CW regime; Effect of the boundary morphology of the sample on the metasurface temperature; Temperature-dependent optical properties of ITO and Au films; Phase shift and reflectance modulation by CdO-based active metasurfaces; Absorbance in the CdO-based active metasurfaces; CdO-based active metasurfaces upon pulsed illumination and toxicity concerns; Properties of the constituent materials; The role of the thermal conductivity the thin SiO_2_ layer on the metasurface temperature: CW regime; Characteristic time scales during pulsed laser illumination; High-irradiance cycling and dynamic beam switching experiments.

## Supplementary Material

Supplementary Material Details
