# Supplementary material for: Electrically tunable conducting oxide metasurfaces for high power applications
Source: Nanophotonics. 2023 Jan 18;12(2):239–53. doi: 10.1515/nanoph-2022-0594 (PMC11501733; doi:10.1515/nanoph-2022-0594)
Supplement: Supplementary file 1 — Supplementary Material Details [file j_nanoph-2022-0594_suppl.docx]

Supplementary Materials

Electrically Tunable Conducting Oxide Metasurfaces for High Power Applications

*Ruzan Sokhoyan^1^, Prachi Thureja^1^, Jared Sisler^1^, Meir Grajower^1^, Komron Shayegan^1^,* *Eyal Feigenbaum^2^, Selim Elhadj^2,3,†^, and Harry A. Atwater^1,^*^*^

^1^Thomas J. Watson Laboratories of Applied Physics, California Institute of Technology, Pasadena, California 91125, USA

^2^National Ignition Facility and Photon Science, Lawrence Livermore National Laboratory, Livermore, California 94550, USA

^3^Materials Engineering Division, Lawrence Livermore National Laboratory, Livermore, California 94550, USA (now at Seurat Technologies, Inc., Wilmington, MA 01887)

**1.** **Link budget analysis and requirements for additive manufacturing**

We determine irradiances a metasurface should be able to withstand to be used in metasurface-based LiDAR and free-space optical communication systems. Figure S1a shows the schematic of the proposed LiDAR system. In the proposed LiDAR system, we shine a light beam onto a metasurface, which then electronically steers the reflected beam. The steered beam scatters off an object, and the scattered light is then detected by the receiver. Unlike the case of LiDAR, in the case of free-space optical communications, the modulated steered beam is directly detected by a remotely placed and tracked receiver (Figure 1b). Hence, the modulated beam steered by the first metasurface is detected by a remotely placed receiver, which is placed in the vicinity of the second beam steering metasurface. And vice versa, a modulated beam steered by the second metasurface is detected by the detector placed in the vicinity of the first metasurface.

We use the LiDAR range equation^1^ to estimate the power at the receiver $P_{receiver}\left( r \right)$ according to

$P_{receiver}\left( r \right)=P_{out}\frac{\rho}{A_{ill}}\frac{A_{rec}}{\pi r^{2}}e^{-2\gamma r}$, (S1)

where $P_{\mathrm{out}}$ is the output power from the metasurface, $A_{ill}$ is the area of the scatterer illuminated by the steered beam, $\rho$ is the scattering cross section of the scatterer (assuming Lambertian scattering), *r* is the distance between the scattering object and the receiver, $A_{rec}$ is the aperture of the collecting lens of the receiver, and $\gamma$ is the attenuation coefficient in the air. We assume that the transmissivity of the receiving optical system is 1. The area of the scatterer illuminated by the steered beam is $A_{ill}=\pi\omega^{2}\left( r \right)=\pi\omega_{0}^{2}\left( 1+\left( \frac{r}{z_{R}} \right)^{2} \right)$, where $\omega_{0}$ is the waist of the beam outcoupled from the LiDAR system, $z_{R}$is the Rayleigh range ($z_{R}=\frac{\pi\omega_{0}^{2}}{\lambda}$) and $r$ is the distance to the target\object, $\lambda$ is the operating wavelength. In these notations, the reflected efficiency from the target towards the detector is$\frac{\rho}{A_{ill}}$. In the case when $r\gg z_{R}$ (*e.g.*, free space optical communication systems), $A_{ill}$can be approximated as $A_{ill}\approx\pi\left( \frac{2\lambda r}{\pi D} \right)^{2}$ . Here, $D$ is the diameter of the laser beam outcoupled from the LiDAR system. In the case when the beam expanding optics is placed in front of the metasurface, $D$ will be defined by the lateral dimensions of this optical component. When no beam expanding optics is used, $D$ is defined by the diameter of the metasurface. The power at the receiver is

$P_{receiver}\left( r \right)=P_{out}\frac{{\rho A}_{rec}}{\omega^{2}\left( r \right)\left( \pi r \right)^{2}}e^{-2\gamma r}=P_{out}\frac{1}{\lambda}\left( \frac{z_{R}}{z_{R}^{2}+r^{2}} \right)\frac{{\rho A}_{rec}}{\pi r^{2}}e^{-2\gamma r}\underset{r\gg z_{R}}{\underbrace{\approx}}P_{out}\left( \frac{D}{2\lambda r} \right)^{2}\frac{{\rho A}_{rec}}{r^{2}}e^{-2\gamma r}$ (S2)

or, in photon number:

$E_{out}\left( r \right)=N_{rec}hc\left( \frac{z_{R}^{2}+r^{2}}{z_{R}} \right)\frac{\pi r^{2}}{{\rho A}_{rec}}e^{2\gamma r}\underset{r\gg z_{R}}{\underbrace{\approx}}N_{rec}hc\lambda\left( \frac{2r}{D} \right)^{2}\frac{r^{2}}{{\rho A}_{rec}}e^{2\gamma r}$, (S3)

$N_{rec}\left( r \right)=E_{out}\frac{1}{hc}\left( \frac{z_{R}}{z_{R}^{2}+r^{2}} \right)\frac{{\rho A}_{rec}}{\pi r^{2}}e^{-2\gamma r}\underset{r\gg z_{R}}{\underbrace{\approx}}E_{out}\frac{1}{hc\lambda}\left( \frac{D}{2r} \right)^{2}\frac{{\rho A}_{rec}}{r^{2}}e^{-2\gamma r}$

where $E_{out}$ is the energy of the outgoing laser pulse, $N_{rec}$ is the number of photons at the receiver, *h* is Planck’s constant, and c is the speed of light. Note that when *γ*=0, for sufficiently distances, $P_{receiver}\propto1/r^{4}$.

First, we analyze the laser power levels required in the case of a metasurface-based LiDAR system. We use the LiDAR range equation, which we now rewrite as:

$P_{receiver}\left( r \right)=R\times P_{in}\frac{1}{\lambda}\left( \frac{z_{R}}{z_{R}^{2}+r^{2}} \right)\frac{{\rho A}_{rec}}{\pi r^{2}}e^{-2\gamma r}$. (S4)

Here, $P_{in}$ is the power of the laser beam impinging on the metasurface, and $R$ is the fraction of the incoming beam energy that has been directed into the steered beam. The distance between the scattering object and the receiver $r$ is assumed to be identical to the distance between the scattering object and the metasurface. As an example, we assume the laser beam is $\omega_{0}=1$mm ($z_{R}\approx2$m, roughly the size of the metasurface), the aperture of the collecting lens at the receiver is $A_{rec}=3 \mathrm{cm}$. Assuming foggy atmospheric conditions, we use the following attenuation coefficient in the air: $\gamma=0.877\left[ \frac{1}{km} \right]$.^1^ In our analysis, we disregard the effects of the atmospheric turbulence on the beam propagation. We assume that only 5% of light scattered off the scatterer reaches the detector. The transmissivity of the receiving optical system is taken to be 100%. We also assume that 22% of the energy of the incoming beam is directed into the steered beam (R=22%). Using the LiDAR range equation, we can now estimate the detection range both in CW and transient illumination regimes.

We identify the incoming powers and pulse energies for which the detection range of the metasurface-based LiDAR system can be 100 m, which is required for autonomous vehicle applications. First, we explore the case of the CW illumination regime. Figure S1c plots the power at the receiver $P_{receiver}\left( r \right)$ as a function of the distance $r$ between the scatterer and the metasurface for different values of the laser beam power $P_{in}$ impinging on the metasurface. In the case of CW illumination, we set the detection threshold of the detector to be 0.01 μW, which is marked by a green dashed line in Figure S1c. As seen in Figure S1c, the detection range of 100 m can be achieved at an incoming power of 0.01 kW.

Note that the incoming power of 0.01 kW corresponds to the irradiance of 0.32 kW/cm^2^. The detection range can be further increased by increasing the irradiance of the laser beam impinging on the metasurface (see Figure S1c). Next, we consider the case when the metasurface is illuminated with a laser pulse. Figure S1d plots the number of photons at the receiver as a function of the distance $r$ between the scatterer and the metasurface for different values of the energy $E_{in}$ of the laser pulse impinging on the metasurface. We assume that the minimal photon number our detector can measure is 100 (see the green dashed line in Figure S1d). In this case, to detect a target at 100 m, the minimal energy of the laser pulse impinging on the metasurface should be 100 nJ. As seen in Figure S1d, the detection range can be further increased by increasing the energy of the incoming laser pulse.

Next, we provide estimates for the scaling of communication distances, which can be attained using the metasurface-based free-space optical communication system for different levels of power handling of the metasurface. We can adapt the LiDAR equation to the case of free-space optical communications by eliminating


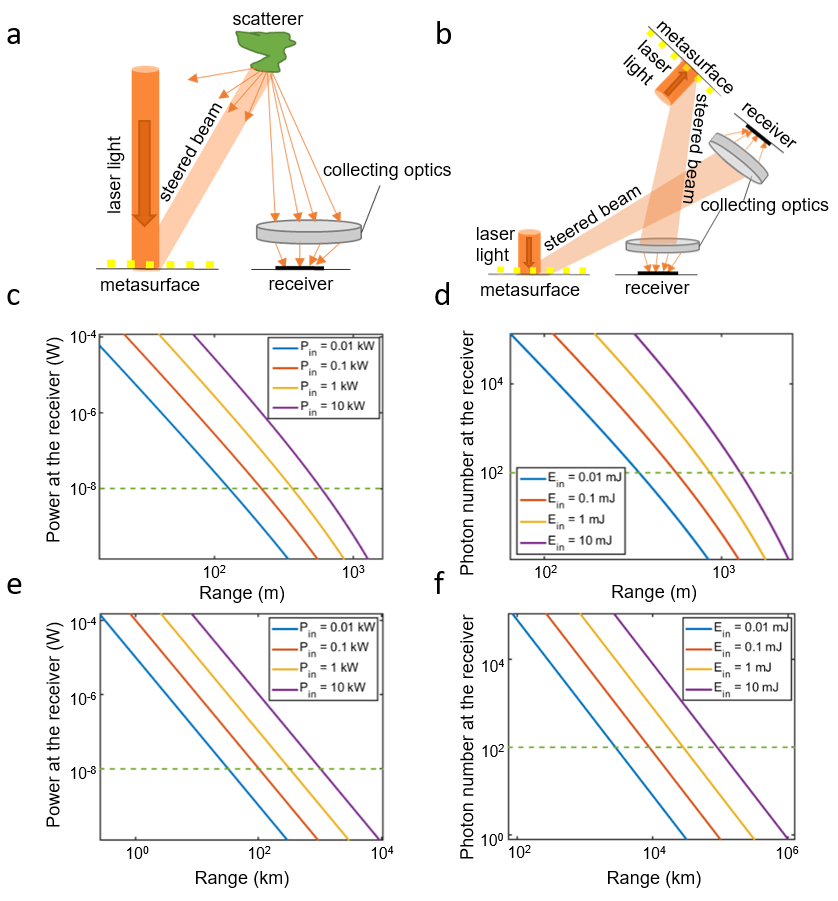


**Figure S1.** a) Schematic of a metasurface-based LiDAR system. b) Schematic of a two-way metasurface-based free-space optical communication system. c) In case of the LiDAR operating in the CW regime, the power at the receiver $P_{receiver}$*vs.* the range from the metasurface for different power values of light impinging on the metasurface $P_{in}$. d) In case of the LiDAR in the pulsed illumination regime, the number of photons at the receiver *vs.* the range from the metasurface for different energies of the laser pulse impinging on the metasurface $E_{in}$. e) In case of free-space optical communications in the CW regime, the power at the receiver $P_{receiver}$*vs.* the distance between the metasurface and the receiver for different power values of light impinging on the metasurface $P_{in}$. f) In case of free-space optical communications in the pulsed illumination regime, the number of photons at the receiver *vs.* the distance between the metasurface and the receiver for different energies of the laser pulses impinging on the metasurface $E_{in}$. In c)-f), the green dashed line denotes the threshold of the receiver. The assumed input beam diameter is 0.2 cm, the diameter of the detector collection area is 3 cm, and the *beam steering efficiency of the metasurface is taken as 22%*.


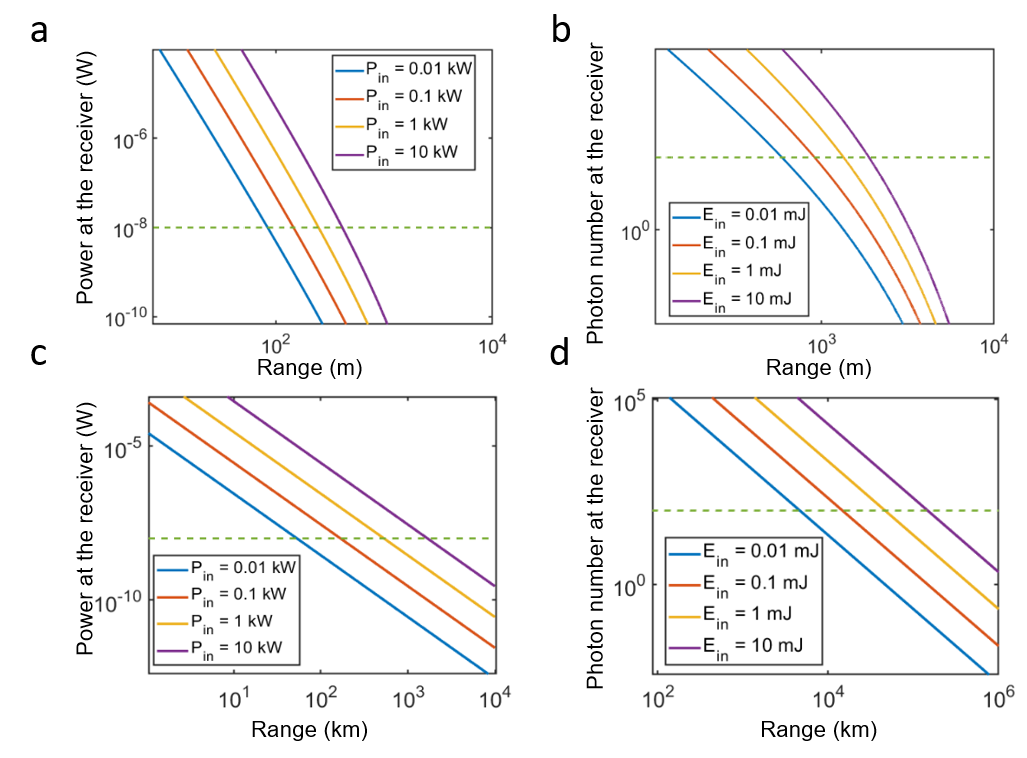


**Figure S2.** a) In case of the LiDAR operating in the CW regime, the power at the receiver $P_{receiver}$*vs*. the range from the metasurface for different power values of light impinging on the metasurface $P_{in}$. b) In case of the LiDAR in the pulsed illumination regime, the number of photons at the receiver *vs*. the range from the metasurface for different energies of the laser pulse impinging on the metasurface $E_{in}$. c) In case of free-space optical communications in the CW regime, the power at the receiver $P_{receiver}$*vs*. the distance between the metasurface and the receiver for different power values of light impinging on the metasurface $P_{in}$. d) In case of free-space optical communications in the pulsed illumination regime, the number of photons at the receiver *vs*. the distance between the metasurface and the receiver for different energies of the laser pulses impinging on the metasurface $E_{in}$. The green dashed line denotes the threshold of the receiver. The assumed beam diameter is 0.2 cm, and the diameter of the detector collection area is 3 cm, and the *assumed beam steering efficiency of the metasurface is 3%*.

the contribution of the scattering object $(\rho=1).$ Within the context of free-space optical communications, $r$ now stands for the distance between the metasurface and the receiver (see Figure S1b). Having space-related applications in mind, we take the attenuation coefficient $\gamma$ to be unity: $\gamma=1$. As in the case of the LiDAR, we assume that the diameter of the incoming beam is $D_{beam}=0.2 \mathrm{cm}$, the aperture of the collecting lens at the receiver is $A_{rec}=3 \mathrm{cm}$, and the beam steering efficiency of the metasurface is 22%. In case of CW illumination, the proposed metasurface-based free-space optical communication system can attain the distance of 100 km for an incoming power of 0.01 kW (Figure S1e). In case of pulsed laser illumination, the proposed metasurface-based free-space optical communication system can attain the distance of 10 000 km for a pulse energy of 0.01 mJ (Figure S1f). In what follows, we will theoretically investigate whether gate-tunable conducting oxide metasurfaces can withstand such laser irradiances or pulse energies, which are of interest for LiDAR and free space optical communications applications.

Apparently, the range of the LiDAR or the communication distance of the free space optical communication system increases with the power or the energy of the incoming laser beam. The power (energy) of the incoming laser beam can be increased by increasing the irradiance (laser fluence) of the metasurface or by increasing the area of the incoming laser beam that implies an increased metasurface area. Another important parameter to consider is the beam steering efficiency of the metasurface, which was set to 22%. For example, if the beam steering efficiency of a metasurface is set to 3%, for a given incoming power (pulse energy) the detection range and communication distance of the metasurface are significantly reduced (Figure S2). Alternatively, the detection range or communication distance can be increased by increasing the aperture of the collecting lens $A_{rec}$.


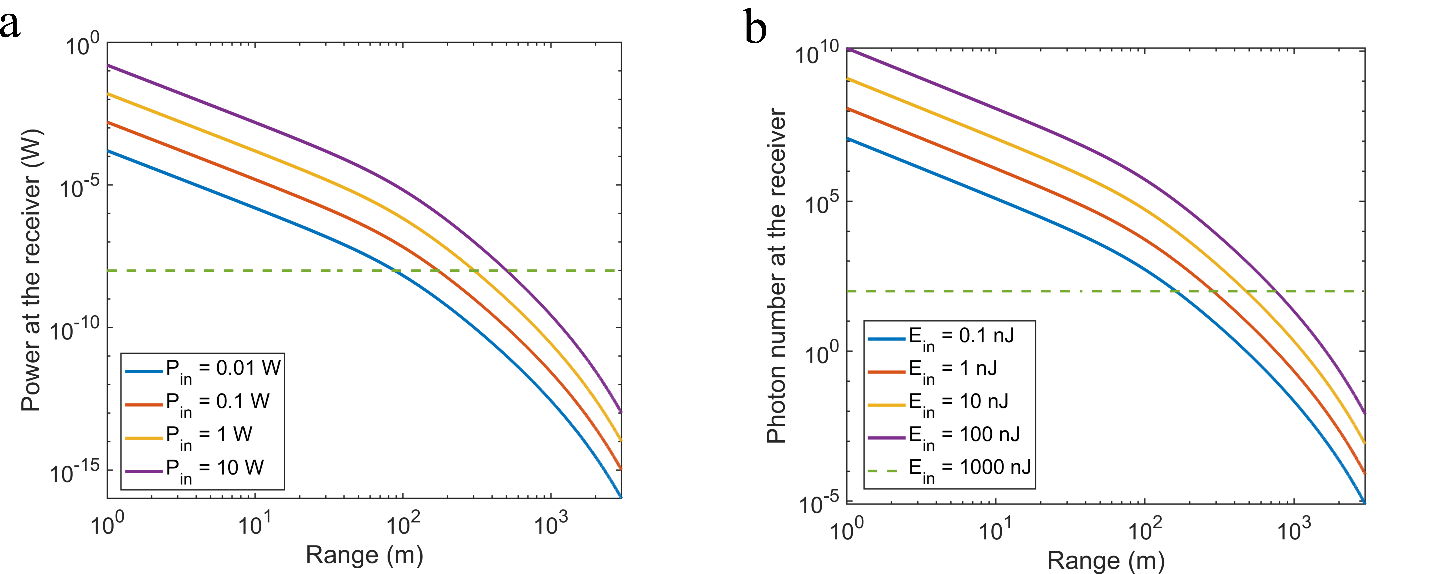


**Figure S3.** a) In case of the LiDAR operating in the CW regime, the power at the receiver $P_{receiver}$*vs*. the range from the metasurface for different power values of light impinging on the metasurface $P_{in}$. b) In case of the LiDAR in the pulsed illumination regime, the number of photons at the receiver *vs*. the range from the metasurface for different energies of the laser pulse impinging on the metasurface $E_{in}$. The assumed beam waist is 0.7 cm, the other parameters are the same as in Figure S1.

In the cases discussed above (Figures S1, S2), the diameter of the steered beam was taken to be 0.2 cm. However, one can increase the diameter of the outcoupled beam by adding additional optical components, which can expand and collimate the steered beam thus reducing the diffraction losses. As an example, we consider the case when the diameter of the laser spot outcoupled from the LiDAR is $\omega_{0}=0.7$ cm ($z_{R}\approx100 m$) (Figure S3). Figure S3 assumes a beam steering efficiency of 3%. Note that in the case of the CW illumination, the power of the incoming light is three orders of magnitude lower as compared with the powers used in Figures S1 and S2. In case of pulsed laser illumination, the incoming pulse energies are five orders of magnitude lower as compared with the ones assumed in Figures S1 and S2. However, due to an optimal choice of the diameter of the outcoupled beam, the proposed LiDAR system is able to efficiently detect objects at target distances exceeding 100 m.

Finally, we briefly discuss requirements of metasurface-based additive manufacturing. In standard additive manufacturing schemes, a focused light beam emitted by a CW laser is mechanically raster scanned on the powder bed. The power of the beam is typically few hundreds of Watts (up to ~1 kW).^2^ This implies, for example, that if the metasurface area is 1 cm × 1 cm, and the metasurface reflectance is ~20%, the irradiance of 5 kW/cm^2^ would yield a 1 kW reflected beam. In the case of a modest reflectance of 3%, the incoming irradiance should be 33 kW/cm^2^, so that the power of the reflected beam is 1 kW. Active metasurfaces can simultaneously electronically steer and focus the reflected beam without employing mechanically moving parts. The ability of metasurfaces to arbitrarily shape the beam could offer interesting opportunities for additive manufacturing. Another important advantage of active metasurfaces is their ability to near-instantaneously change the position of the laser spot, which would impact the beam dwell time and temperature history and profile thus altering the powder melting dynamics and residual stresses. In contrast, galvanometric mirrors, which change the direction of the beam in current additive manufacturing schemes, cannot change the beam direction in such near-instantaneous fashion. For example, typical fast galvanometric mirrors with an aperture of 5 cm will require 5 ms to shift the beam by 10 mrad. Prior research has demonstrated ITO-based optical modulators operating at GHz frequencies.^3^ Thus, in principle, active metasurfaces operating at such high modulation frequencies (or a least at modulation frequencies of ~100 MHz) could also be achievable.

**2. Electrical and optical properties of ITO and CdO**

Simple physics consideration can set an upper bound on the carrier density in the active ITO layer. Let us consider a planar structure, which consists of Au, HAOL, and ITO (Au/HAOL/ITO) in which Au and ITO electrodes are biased with respect to each other (for the considered voltage polarity see Fig. 1a). Let us also assume that the thickness of the charge accumulation layer in ITO is *t_ac_*, and the charge density in this accumulation layer is constant. The assumed thicknesses of the dielectric HAOL layer is *t_d_*. In this case, using Gauss’s law we can derive the following relation between the carrier density increase in the accumulation layer Δ*N* and applied gating voltage *V_g_*: Δ*N = ɛ_0_ k_d_ V_g_/(e t_ac_ t_d_).* This relation was derived by considering a parallel plate capacitor structure and integrating over a cylindrical surface such that the axis of the cylinder is perpendicular to the capacitor plates, and the considered cylinder also encompasses one of the capacitor plates. Here, $\varepsilon_{0}$ is the dielectric permittivity of vacuum, *e* is the electron charge, *k_d_* is the DC permittivity of the gate dielectric, which is HAOL in our case. In our metasurface design, the thickness of the HAOL layer is *t_d_ =* 10 nm. Prior research has reported that the DC permittivity of HAOL is *k_d_* = 22 while the breakdown field in HAOL is 0.72 V/nm, which, in our case, corresponds to the maximal applied voltage of 7.2 V.^4^ If the assumed thickness of the accumulation layer is *t_acc_ =* 1 nm^5^, then the carrier density in the accumulation layer *N_acc_* can vary between the background carrier density *N_bg_* and *N_bg_+*8.8×10^20^ cm^-3^, as calculated from the equation for Δ*N* above. Thus, we conclude that when the considered gate dielectric is 10 nm-thick HAOL, the carrier density in the 1 nm-thick active ITO layer can be maximally increased by 8.8×10^20^ cm^-3^.

The complex dielectric permittivity of the ITO layer can be described by the Drude model: $\varepsilon_{ITO}=\varepsilon_{\infty}-\frac{\omega_{p}^{2}}{(\omega^{2}+i\omega\Gamma)}$. Here, $\omega$ is the angular frequency of light and $\Gamma$ is the damping constant. The plasma frequency $\omega_{p}$ is related to the ITO charge carrier density *N* and the effective electron mass $m^{*}$ according to $\omega_{p}=\sqrt{\frac{Ne^{2}}{(\varepsilon_{0}m^{*})}}$. In our simulations, we use $\Gamma$ =1.8×10^14^ rad×Hz, *m** = 0.35 *m_e_*, and $\varepsilon_{\infty}$=3.9, where *m_e_* is the free electron mass.^18^ We also assume the bulk electron density of ITO is *N*=3×10^20^ cm^-3^.

As in the case of ITO, the complex dielectric permittivity of cadmium oxide (CdO) *ɛ*_CdO_ can also be described by the Drude model with the following Drude parameters: $\Gamma$ =3.3×10^13^ rad×Hz, *m** = 0.21 *m_e_*, $\varepsilon_{\infty}$=5.5.^6^

**3.** **Phase shift and reflectance modulation by ITO-based active metasurfaces**


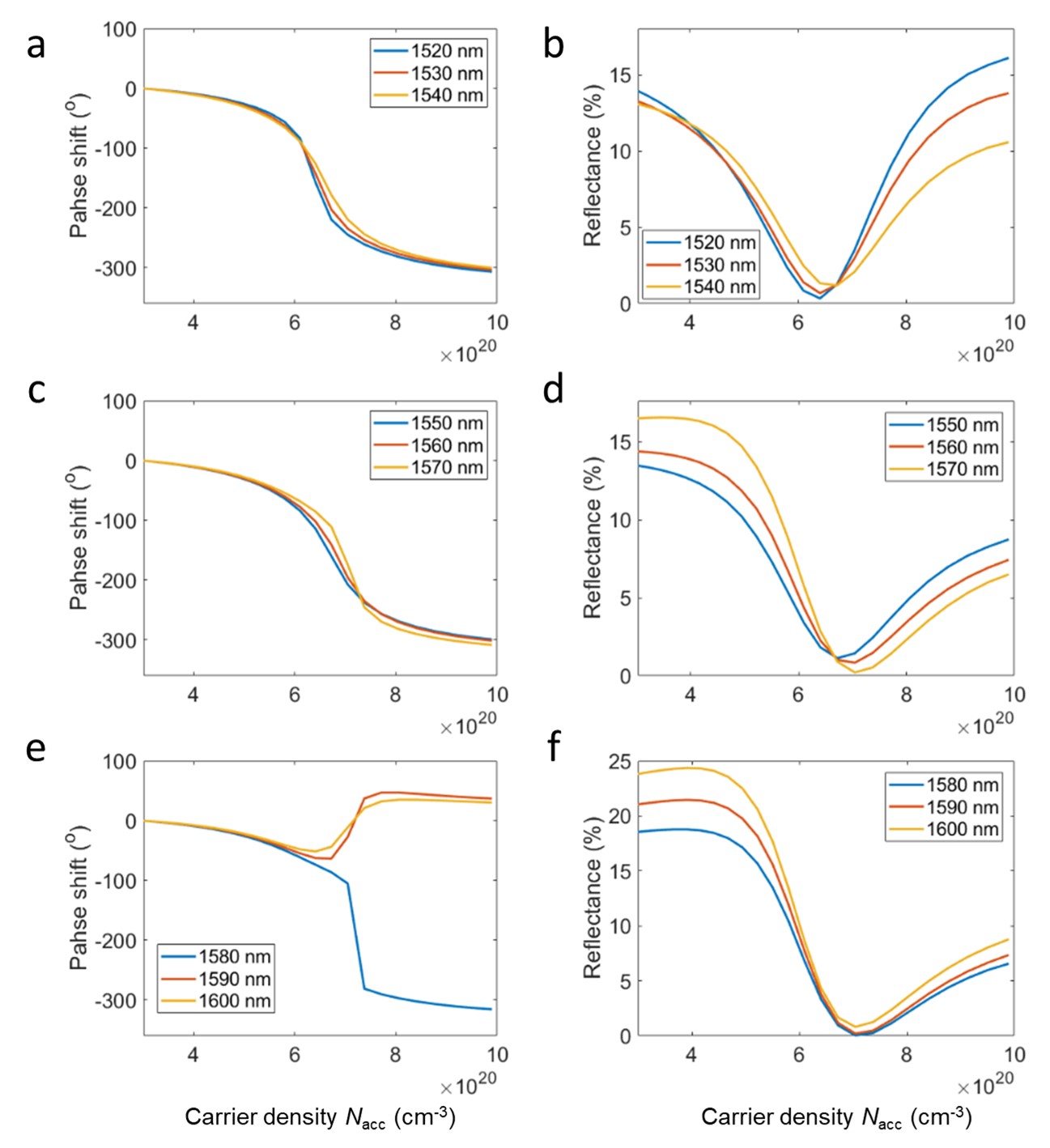


**Figure S4.** Electrically tunable optical response of the ITO-based gate-tunable metasurface schematically depicted in Fig. 1a. We plot the phase shift and the reflectance as a function of the carrier density of the active ITO layer for different fixed values of the operating wavelength. Legends indicate the values of the operating wavelengths in each case.

**4. Absorbance in ITO-based active metasurfaces**


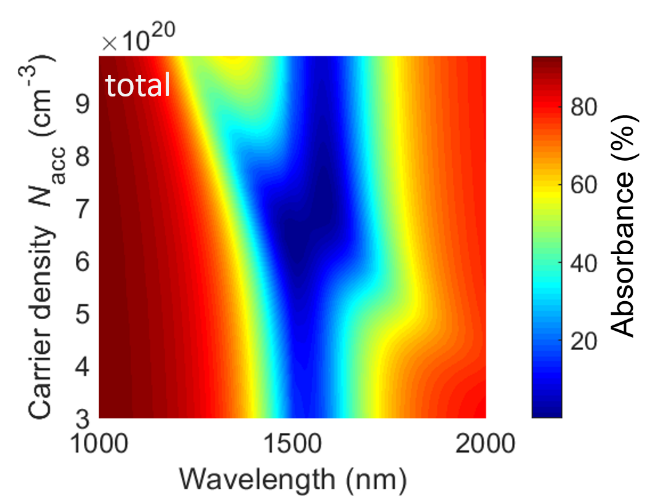


**Figure S5.** a) Absorbance in ITO-based active metasurface as a function of wavelength and the carrier density in the active ITO layer.


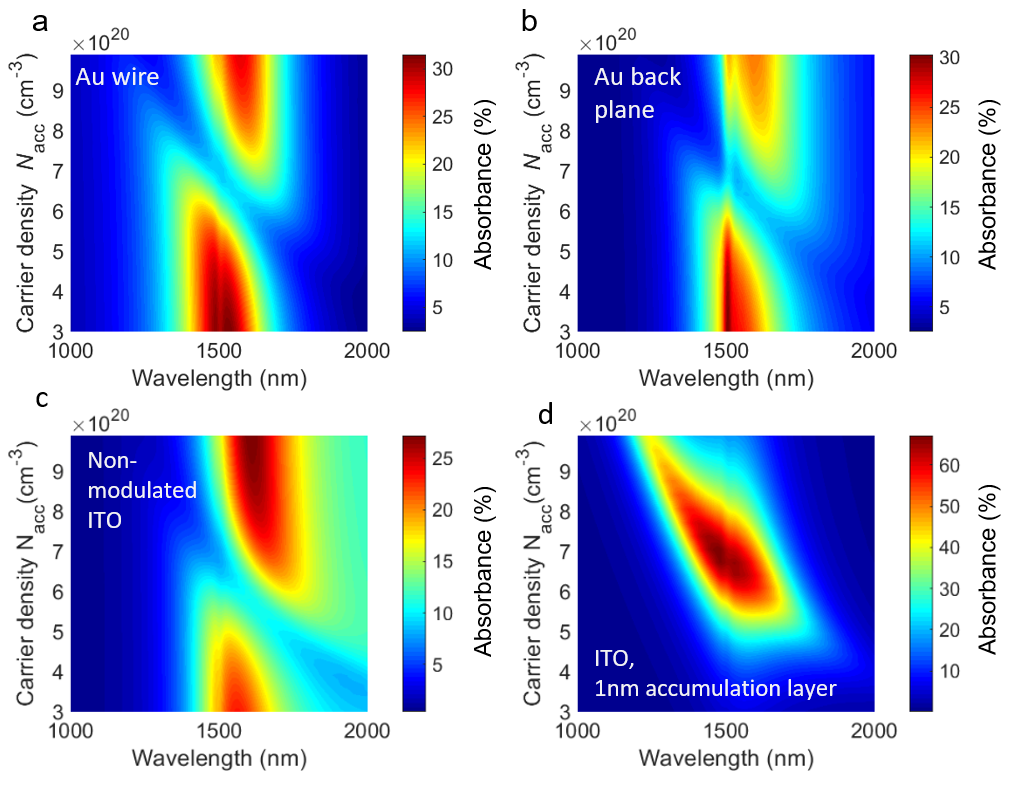


**Figure S6.** Absorbance in different constituent layers of ITO-based active metasurface as a function of wavelength and the carrier density in the active ITO layer. Absorbance in a) the Au wire, b) the Au back plane, c) 4 nm-thick non-modulated ITO layer, d) 1 nm-thick ITO accumulation layer.

**5. Spatial distribution of the electric field inside ITO-based active metasurfaces**


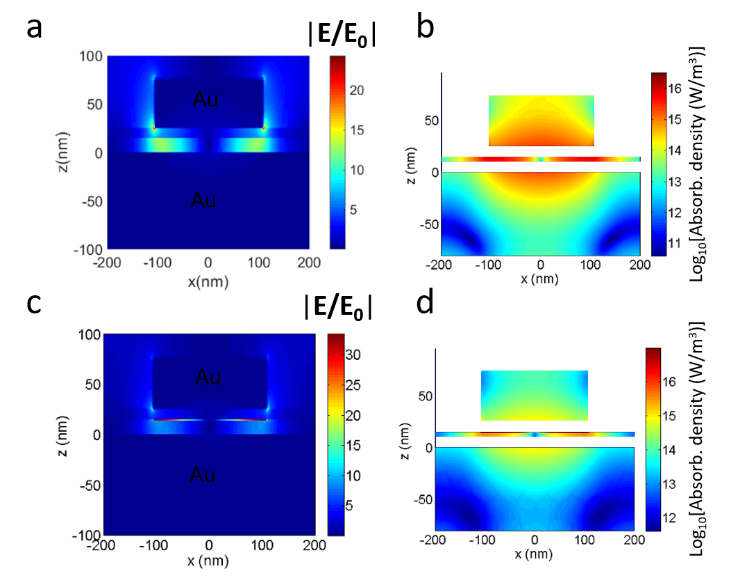


**Figure S7.** a) Spatial distribution of the electric field |**E**| inside the ITO-based active metasurface at no applied bias. b) Power absorbed per unit volume (absorption density) in the metasurface unit cell at zero applied bias and for an irradiance of 3.5 kW/cm^2^. c) Spatial distribution of the electric field |**E**| inside the ITO-based active metasurface when the accumulation layer of ITO is in the ENZ regime (*N*_acc_=6.5×10^20^ cm^-3^). d) Spatial distribution of the electric field |**E**| inside the metasurface when the accumulation layer of ITO is in the ENZ regime (*N*_acc_=6.5×10^20^ cm^-3^).

Figure S7 plots the spatial distribution of the optical electric field inside the metasurface as well as the calculated absorption density. In the case when no electrical bias is applied, we observe that the electric field is concentrated in the dielectric spacer between the Au wire and the Au back reflector (Figure S7a). Figure S7b plots the calculated absorption density in the case without applied bias. Note that we left blank the spatial areas in which the lossless (non-absorbant) HAOL and SiO_2_ layers are located. The calculated absorption density exhibits significant enhancement in the ITO layer as well as at the bottom surface of the Au wire and at the top surface of the Au back reflector. When the ITO accumulation layer is in the ENZ regime, we observe a strong optical electrical field enhancement in the 1-nm–thick active layer (Figure S7c). The calculated absorption density also exhibits enhancement in the 1-nm–thick ITO accumulation layer (Figure S7d).

**6. Thermal behavior of ITO-based active metasurfaces: CW regime**

Next, we identify the key optical and material parameters which drive the temperature rise in the metasurface during CW illumination. Reducing the amount of the absorbed power will, clearly, reduce the temperature rise of the metasurface. To further explore the effect of the reduced absorptance, we plot the peak temperature in the metasurface as a function of the wavelength of the incoming light with an irradiance


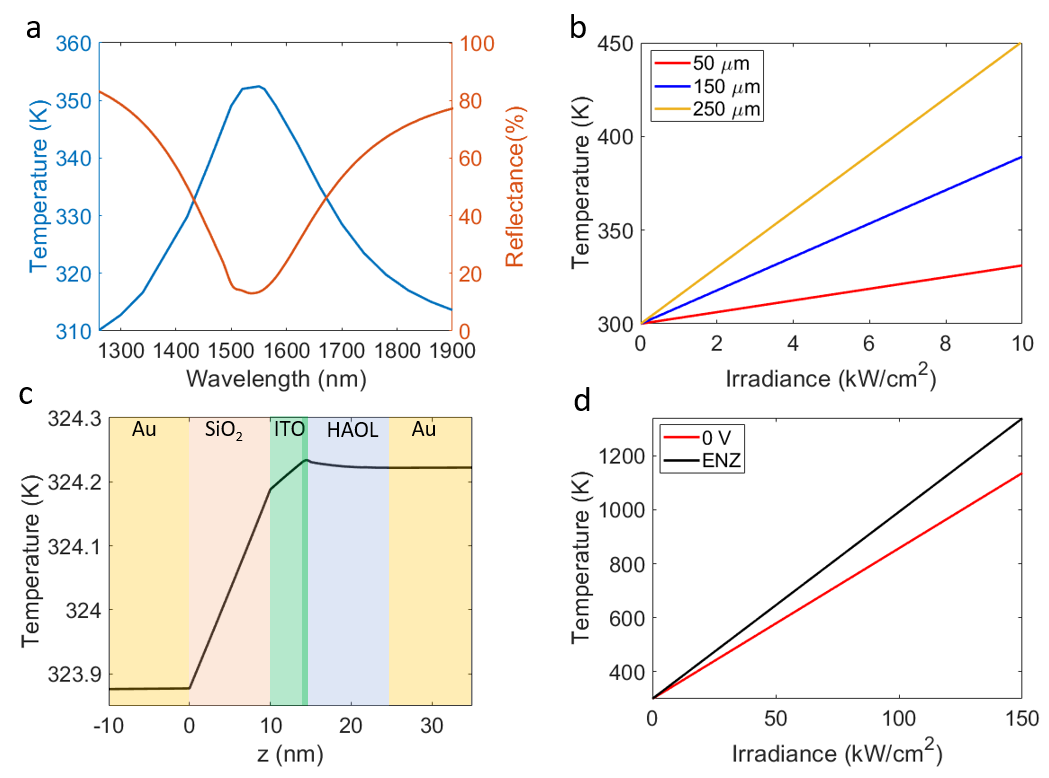


**Figure S8.** Thermal response of the ITO-based gate-tunable metasurfaces upon CW illumination at a wavelength of 1550 nm. In a), the red curve shows the reflectance spectrum of our metasurface at no applied bias. In a), the blue curve shows the peak temperature attained in the metasurface for the thickness of the Si substrate of 250 μm and the irradiance of 3.5 kW/cm^2^. b) Peak temperature in the metasurface as a function of the irradiance for different thicknesses of the Si substrate. c) Temperature distribution inside the metasurface unit cell when *x* is fixed as *x*=100 nm (dark green layer indicating the 1-nm–thick active accumulation layer). In c), the substrate thickness is taken as 100 μm. d) Peak temperature inside the metasurface as a function of the laser irradiance. The assumed substrate thickness is 100 μm. In d), the red curve corresponds to the case of no applied bias while the black curve corresponds to the case when the metasurface is biased so that the ITO accumulation layer is in the ENZ regime (*N_acc_*=6.5×10^20^ cm^-3^).

of 3.5 kW/cm^2^ (Figure S8a). In Figure 4a, the thickness of the Si is taken as 250 μm. As the operating wavelength moves away from the resonant wavelength, for a given bulk charge density without applied bias, the reflectance increases. As expected, the increase in the reflectance is accompanied by a decrease in the temperature. We observe that, at the resonant wavelengths, the peak temperature in the metasurface is around 350 K. On the other hand, at an off-resonant wavelength of 1250 nm, when the metasurface reflects over 80% of the incoming light, the peak temperature attainable in the metasurface is reduced to 310 K. Therefore, an improved active metasurface design, which exhibits higher reflectance while maintaining tunable optical properties, would significantly increase the laser power the metasurface can withstand.

The choice of the substrate can also strongly affect the thermal response of the metasurface. When investigating the effect of the operating wavelength, we took the baseline thickness of the Si substrate to be 250 μm. However, changing substrate thickness can strongly affect the peak temperature of the metasurface during illumination.^7-8^ Figure S8b plots the peak temperature of the metasurface for different thicknesses of the Si substrate as a function of laser irradiance. For a fixed substrate thickness, the peak temperature is a linear function of the laser irradiance. This result is consistent with prior experimental reports in the literature.^9-11^ As seen in Figure S8b, the peak temperature in the metasurface can be dramatically reduced when reducing the substrate thickness (Figure S8b). This strong dependence on the substrate thickness can be intuitively understood by considering heat flow in a solid kept at two different temperatures at two ends, *T*_1_ and *T*_2_, respectively. In this case, the amount of heat flowing through the unit area of the solid *Q* can be written as *Q = k (T*_2_*-T*_1_*)/L*. Here, *L* is the length of the solid, and *k* is the thermal conductivity of the solid. The presented relation shows that reducing the solid length would increase the heat flow. If we assume that the temperature at the hot side of the solid is not fixed, this increased heat flow will result in a decrease of the peak temperature in the solid. This relation also indicates that increasing the thermal conductivity of the substrate would also decrease the peak temperature attainable in the metasurface, which our modeling captures consistently, as expected. Thus, while the optical properties of the metasurface are not affected by the choice of the substrate, choosing a thinner substrate with higher thermal conductivity would significantly increase the laser damage threshold of the metasurface because the heat flux to the heatsink is increased due to a steeper temperature gradient across a thinner substrate.

Next, we examine the spatial variation of temperature inside the metasurface in the case when the active ITO layer is in the ENZ regime (*N*_acc_=6.5×10^20^ cm^-3^). Figure S8c plots the temperature inside the metasurface along *z* direction (depth of the heterostructure) at a fixed value of *x* coordinate: *x* = 100 nm Figure S8a). In other words, the considered *z*-line is 8 nm away from the edge of the Au wire. In our notation, the plane *z*=0 corresponds to the interface of the back reflector and the SiO_2_ layer, and the top wire is located from *z*=25 nm to *z*=75 nm (see also Figure S8a). The thickness of the Si substrate is 100 μm, and the irradiance is, again, 3.5 kW/cm^2^, the same irradiance value as in the case of no applied voltage. As seen in Figure S8c, we now reach the highest temperature in the active ITO layer (dark green region). However, despite the fact that the absorption density in the active ITO layer is significantly enhanced, the temperature increase in the accumulation layer with respect to the temperature of the other layers in the metasurface structure is practically negligible (<1 K).

We now focus on the peak temperature increase in the metasurface as a function of the laser irradiance under CW illumination for the cases of both i) no applied voltage (0 V) and ii) when the active ITO layer is in the ENZ regime (*N*_acc_=6.5×10^20^ cm^-3^). The thickness of the Si substrate is taken to be 100 μm. At the lower end of the laser irradiance, the temperature increase due to the applied bias is very limited. However, this temperature increase becomes significant at higher laser irradiances. We observe, that at a laser irradiance of 150 kW/cm^2^, the peak temperature of the metasurface equals the melting temperature of Au provided that the heat sink temperature can be maintained at 300 K. For the purposes of this discussion, this can be viewed as a theoretical upper bound of the laser irradiance that the metasurface can support under applied voltage, however in practice the melting breakdown would likely be much lower. In the case of no applied bias, the melting temperature of Au is reached at an irradiance of 187 kW/cm^2^, an irradiance level tolerated by the device that is 18% higher than when in active switching conditions based on melting failure criteria. We would like to emphasize that in the cases of both CW illumination and transient illumination with a few ns-long laser pulses, the pre-dominant mode of material damage is related to thermal heating and melting of materials.^12^ In practice, we expect that active semiconductor-based metasurface devices should exhibit an adequate electronic performance at least for temperatures below 400 K. ^13^ For the substrate thickness of 100 μm, the metasurface temperature of 400 K corresponds to the laser irradiance to 14.6 kW/cm^2^. Note that the ITO metasurface-based LiDAR system attains a detection range of ~100 m at an incoming power of 0.01 kW. The incoming power of 0.01 kW corresponds to an irradiance value of 0.3 kW/cm^2^, which yields a modest temperature increase at any of the considered substrate thicknesses (Figure S8b). Hence, our ITO-based active metasurface is expected to easily meet LiDAR applications requirements from a laser damage perspective.

**7. Effect of the boundary morphology of the sample on the metasurface temperature**

We can also use simple considerations to estimate metasurface temperatures in case of different sample morphologies. The areas of the ITO-based active metasurfaces previously demonstrated by our group were 40 µm × 40 µm.^14-15^ The area of the substrate, which is covered by an Au, gate dielectric, and ITO layers, may significantly exceed the area of the metasurface. Since only the metasurface area is optically heated, the heat generated in the metasurface will be conducted to the sides of the chip, resulting in the metasurface cooling. On the other hand, in our optical and thermal simulations, we assume insulating boundary conditions in *x* direction, and the structure is assumed to be infinite in *y* direction. Also, in our simulations, we fix the temperature at the bottom of the substrate to 300 K, assuming that the sample is placed on a thermostat. However, we can estimate the temperature of the metasurface accounting for its final dimensions and assuming that no thermostat is available.

To estimate the temperature of the metasurface on a semi-infinite substrate, we use previously developed methodology.^9, 16^ The approach assumes that the metasurface is illuminated by a Gaussian beam of a diameter *D*, which heats the metasurface locally. We can then describe this scenario as a heated disc, which is placed on an infinite Si substrate^9^. The temperature increase *ΔT* of the metasurface is then related to the generated heat *q* according to *q=S k ΔT*, where *k* is the thermal conductivity of the substrate while *S=2D* is the shape factor of the disc with *D* being the diameter of the heating beam^16^. Our experimental sample is fabricated on a Si substrate, which is covered by a 1 μm-thick layer of SiO_2_. To derive an upper bound on the temperature attainable by the sample, we first derive temperature estimates for the case when the sample is placed on an SiO_2_ substrate (see the insets of Fig. S9). When the metasurface is built on an SiO_2_ substrate, for an irradiance of 9.1 kW/cm^2^, an absorbance of 99.3%, and a laser spot diameter of 8 μm, we estimate that the temperature increase of the metasurface is 167 K. On the other hand, if we consider the case of a Si substrate with a thermal conductivity of *k*=148 W/(m K), for an irradiance of 3.5 kW/cm^2^, an absorbance of 90%, and the laser spot diameter of 40 μm, the temperature increase of the metasurface is only 3.3 K. This illustrates how the thermal conductivity of the substrate affects the temperature attained by the metasurface.

In what follows, we derive few simple estimates for the temperature increase of the metasurface *on a Si substrate* using the simple model described above. The model projects that at an irradiance of 567 kW/cm^2^, absorbance of 85%, and for a laser spot diameter of 8 μm, the temperature increase of the metasurface is ~102 K. For the high irradiance of 567 kW/cm^2^, the temperature increase is limited to only 102 K due to the heat conduction in the lateral (in plane) directions and through the heat sink out of plane.


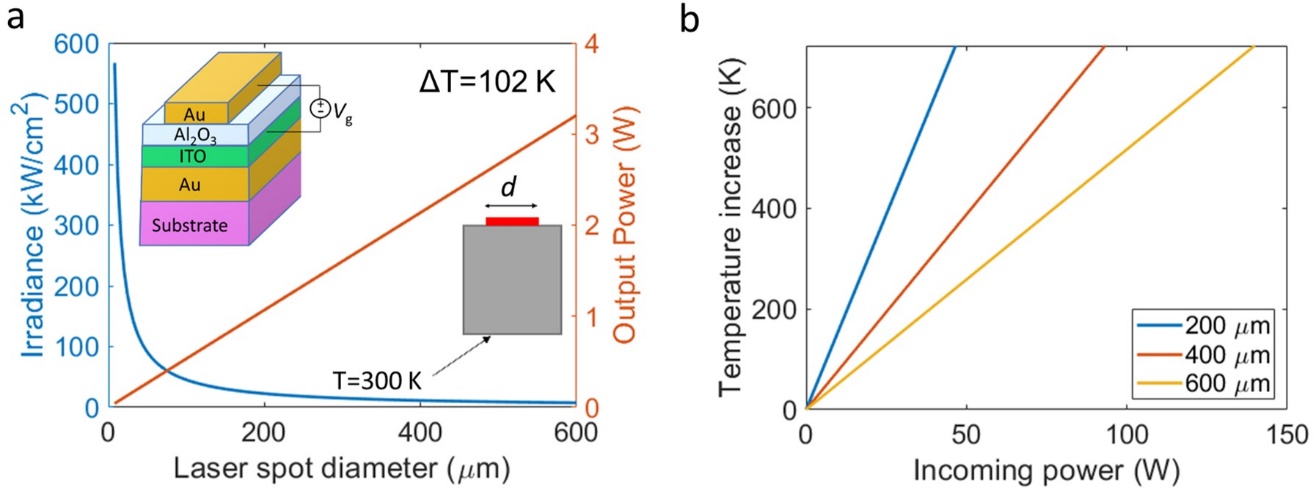


**Figure S9.** Modelled temperature increase upon small-area CW illumination. a) Irradiance, which yields a temperature increase of ΔT=102 K, as a function of the laser spot diameter (red curve). The corresponding total output power is plotted in red. In a) and b), we assume that the metasurface illuminated by a focused laser spot can thermally be modelled as a heated disc on an infinite substrate, which is schematically shown in the inset of a). Schematic of the fabricated metasurface unit cell is also shown in the inset of a). b) The calculated temperature increase of a metasurface as a function of the incoming power for different diameters of the heated disk.

Further, we calculate the laser irradiance, which yields the same temperature increase of 102 K at different diameters of the illuminating laser spot size (Figure S9a). While the area of the fabricated metasurface is 40 μm × 40 μm, in our model, we also consider the cases of larger metasurfaces and extend the diameter of the laser spot up to 600 μm at fixed irradiance of 567 kW/cm^2^. As seen in Figure S9a, the irradiance yielding a temperature increase of 102 K decreases with the laser spot diameter. This is because at larger diameters of the heated spot, the lateral conduction of heat is less efficient. On the other hand, the overall incident power increases with the laser diameter exceeding 3W at the laser spot diameter of 600 μm. Using the same simple relation (*q=S k ΔT*), we calculate the temperature increase of the metasurface as a function of the incoming power for different laser spot diameters (Figure S9b). The temperature range displayed in Figure S9b is limited to 723 K, which corresponds to the melting temperature of thin Au films.^17^ As seen in Figure S9b, to achieve a given temperature increase, larger metasurfaces should be illuminated by laser beams of higher power. These higher laser powers would, however, correspond to lower irradiances (Figure S9a).

We also used a more elaborate model to estimate the temperature increase in our metasurfaces.^18^ The developed model describes a situation when a heated disc is placed on a finite substrate (Figure S10). Again, the metasurface acts as a heat source with an assumed irradiance of 3.5 kW/cm^2^ and an assumed absorbance of 90%. We assume that *S_1_*=40 μm, *t*=2.5 mm, the convective coefficient at the bottom of the substrate is *h*=10 W/(m^2^K), which corresponds to the case of the unforced convection. The thermal conductivity of the Si substrate is k=148 W/(m K). Figure S10c plots the temperature increase of the metasurface as a function of the substrate size S_2_. As seen in Figure S10c, the temperature of the metasurface decreases with the substrate size. For example, for an 8 cm-wide substrate, the temperature increase is ~ 8K, while for a 10 cm-wide substrate the temperature increase is about 5.5 K. Hence, for reasonably large substrates, the projected temperature increase in our metasurface can be quite modest even without introducing a thermostat. Note that the developed model describes the case when there is no forced convection in the system.

The simulation methodology employed in our work has been previously used to accurately reproduce experimental results in the transient illumination regime. We have previously performed transient heat simulations for thin planar films illuminated by ns-long laser pulses.^12^ For the experimentally used laser damage fluences, the simulated temperatures of thin Au films were very close to the previously measured melting temperatures of Au films.^17^

Additionally, we simulated the temperature of one, three, and five metasurface elements placed on a planar heterostructure when the temperature at the bottom of the substrate is fixed to T=300 K. Figures S11a, S11b, and S11c correspond to the cases when one, three or five metasurface elements act as a heat source, respectively. Since we fix the irradiance in our optical simulation, the total input power in the performed simulations will be proportional to the number of the metasurface elements. Importantly, we observe that the temperature of the metasurface elements is much lower as compared with the case when the simulation period is set to 400 nm. We also observe that the temperature of the metasurface elements decreases when increasing the simulation width. Both trends are due to the heat conduction in the lateral direction.


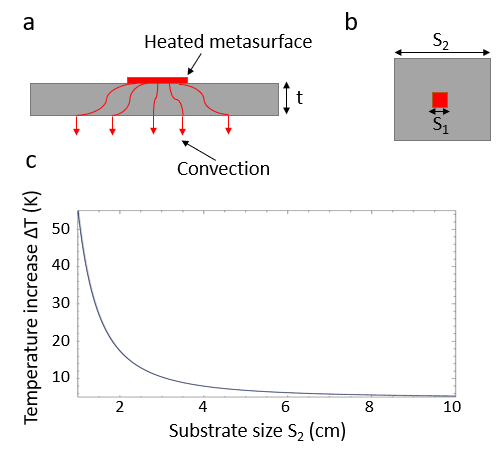


**Figure S10.** Schematic of a heated metasurface on a substrate. a) Side view. b) Top view. c) Calculated temperature increase as a function of the substrate size S_2_. We assume that *S_1_*=40 μm and *t*=2.5 mm. The assumed irradiance is 3.5 kW/cm^2^, and the assumed absorbance is 90%.

**
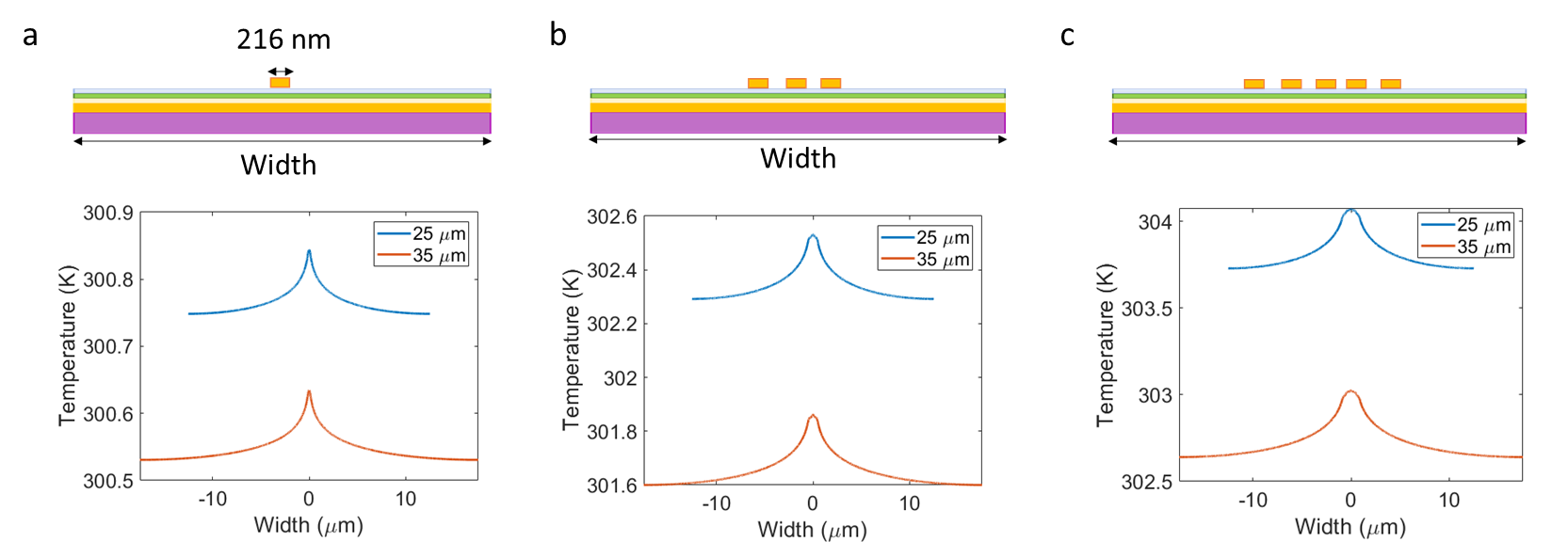
**

**Figure S11.** The top panels of a), b), and c) show the schematics of the considered structures, which consist of a bottom heterostructure section of the metasurface with either one a), three b) or five c) stripe antennas on top. The bottom panels of a), b), and c) plot the temperature in the ITO layer as a function of the *x* coordinate for different periods of thermal simulation. The utilized absorption density is the same as in Fig. S7b and corresponds to the irradiance of 3.5 kW/cm^2^.

In summary, the temperature of the metasurface will depend on the details of the thermal packaging (especially in the CW illumination regime), and no universal solution is possible. However, the configuration considered in our work is a canonical example of photothermal heating of electrically tunable active metasurfaces. Importantly, our numerical analysis carefully accounts for the details of the spatial distribution of the absorption density inside the metasurface. We carefully account for the thermal properties of the constituent material layers as well. Thus, our analysis can serve as a foundation for the evaluation of different sample morphologies.

**8.** **Temperature-dependent optical properties of ITO and Au films**

We use spectroscopic ellipsometry to measure temperature-dependent optical constants of thin planar material films, which constitute ITO-based active metasurfaces. We measured the following samples:

a) A Si substrate;

b) An 8.3 nm-thick Al_2_O_3_ film on a Si substrate;

c) An 80 nm-thick Au film on a Si substrate;

d) A 16.7 nm-thick ITO film on a Si substrate;

e) A planar heterostructure Si(substrate)/Au(80 nm)/ITO(16.7 nm)/ Al_2_O_3_(8.3 nm);

In our samples, ITO films were deposited *via* RF sputtering, Au films – *via* electron beam evaporation, while Al_2_O_3_ layers were deposited *via* atomic layer deposition (ALD). All identical layers were deposited simultaneously.

We placed our samples on the Linkam heating stage, which was mounted onto the ellipsometer setup (Woollam-VASE UV-VIS). Our samples were exposed to the ambient during the measurement. We performed two heating cycles for each sample. First, we performed measurements at temperatures of 300 K, 323 K, 373 K, and 423 K. Then we waited until the sample cooled down to room temperature and remeasured it at 300 K, 323 K, 373 K, and 423 K. In what follows, we refer to these two temperature cycles as *the first temperature cycle* and *the second temperature cycle*.


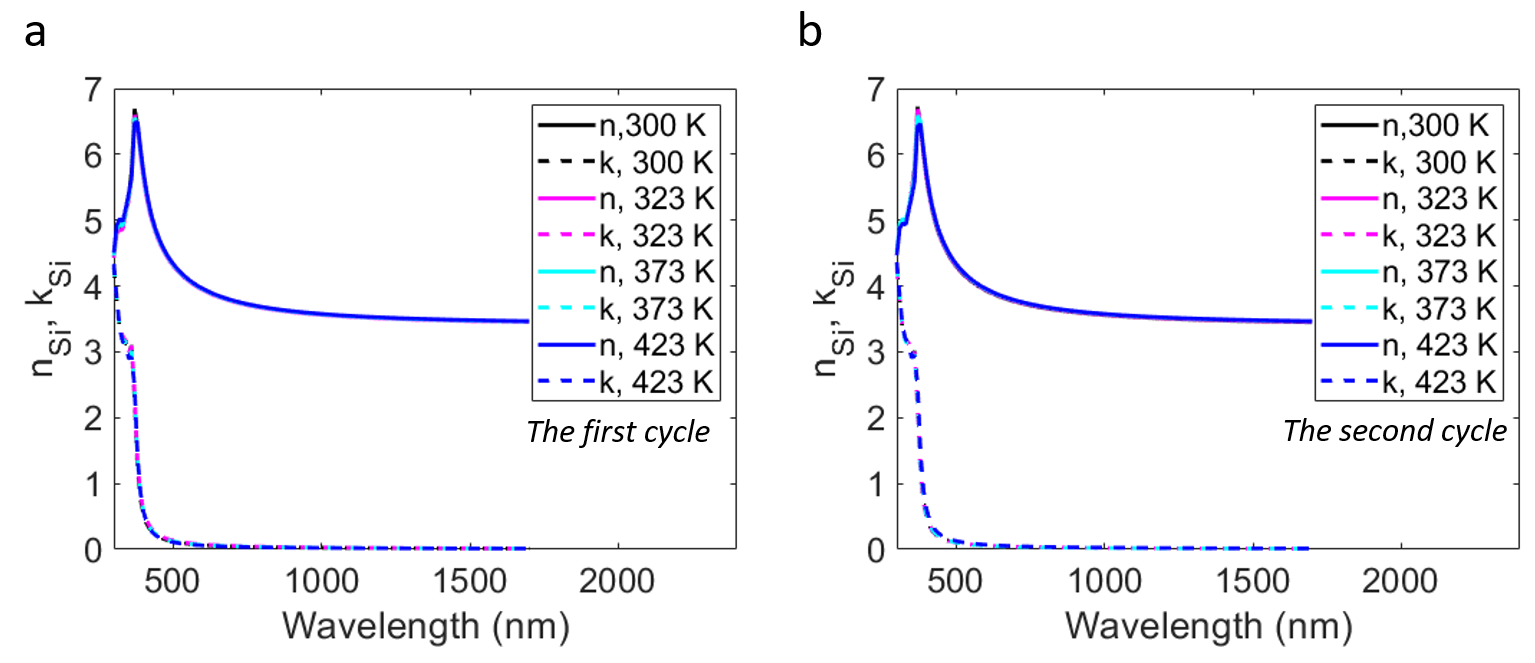


**Figure S12.** Ellipsometry measurements of the real (n_Si_) and imaginary (k_Si_) parts of the complex refractive index of the Si substrate at different temperatures. a) The first temperature cycle. b) The second temperature cycle.

First, we characterize a Si substrate and extract its temperature-dependent complex refractive index. The complex dielectric permittivity of Si is described as a sum of two Lorentz oscillators and one Tauc-Lorentz oscillator. We observe a slight red shift of the real part of the refractive index n_Si_ with increased temperature as well as a slight reduction of n_Si_ at wavelengths around 370 nm (Figure S12). However, in the NIR wavelength range, the temperature-dependent variation of the refractive index is not significant. These results are consistent with the previous reports in the literature^19^. The amount of the complex refractive index variation observed in both temperature cycles is approximately the same. Importantly, for all following fits, we account for the temperature-dependent dielectric permittivity of Si.

Next, we measured the sample, which is comprised of an 8.3 nm-thick Al_2_O_3_ film on a Si substrate. Our measurements show that the optical properties of the Al_2_O_3_ film do not change when increasing the temperature.

The results of the ellipsometry measurements performed on an 80 nm-thick Au film on a Si substrate are summarized in Figure S13. We observe a slight variation of the real part of the dielectric permittivity of Au during the first temperature cycle (Figure S13a). We also observe that the imaginary part of the dielectric permittivity of Au increases during the first temperature cycle (Figure S13b). During the second temperature cycle the variation of the real part of the dielectric permittivity of Au Re(ε_Au_) somewhat decreases (Figure S13b). Figure S13c shows that also in the second temperature cycle, Im(ε_Au_) increases with temperature. Similar trends have already been previously observed.^17^

Next, we extract the dielectric permittivity of a 16.7 nm-thick ITO film on a Si substrate (Figure S14). The dielectric permittivity of ITO was described as a sum of a Lorentz oscillator and a Drude oscillator. We observe that the real part of the dielectric permittivity of ITO Re(ε_ITO_) exhibits a significant variation during the first heating cycle. However, during the second heating cycle, the variation of Re(ε_ITO_) is modest. Moreover, we observe that in the NIR wavelength range, Re(ε_ITO_) extracted from the second heating cycle is larger when compared to Re(ε_ITO_) extracted from the first heating cycle. For example, in the first cycle, at 300 K and at a wavelength of 1550 nm, Re(ε_ITO_)=1.8. In the second cycle, at 300 K, Re(ε_ITO_)=2.5. Thus, the ITO film undergoes an irreversible change when heated by ΔT=123 K.


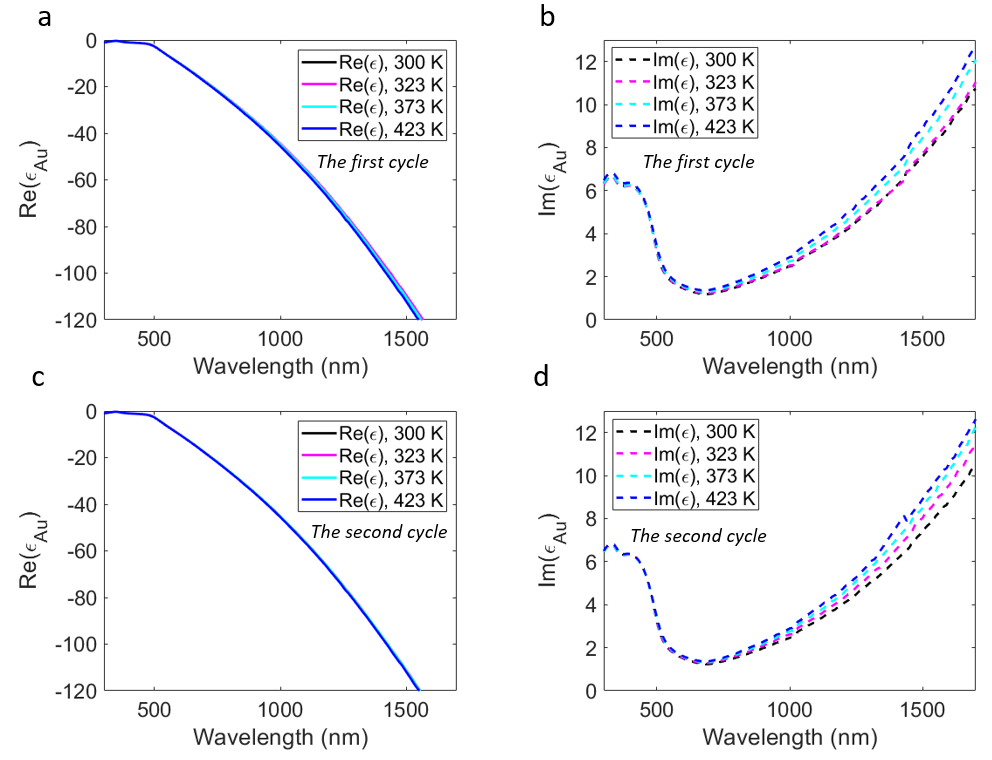


**Figure S13.** Measured real (Re(ε_Au_)) and imaginary (Im(ε_Au_)) parts of the complex dielectric permittivity of an 80 nm-thick Au film at different temperatures. a) and b) correspond to the first temperature cycle while c) and d) correspond to the second temperature cycle.

| Sample and the measurement condition | $\varepsilon_{\infty}$ | *N* (cm^-3^) | *µ* (cm^2^/(V⋅s)) |
| --- | --- | --- | --- |
| Si/ITO, 300 K, cycle 1 | 3.97 | 3.7×10^20^ | 19.4 |
| Si/ITO, 423 K, cycle 1 | 3.78 | 2.8×10^20^ | 17.5 |
| Si/ITO, 423 K, cycle 2 | 3.94 | 2.6×10^20^ | 12.7 |
| Si/Au/ITO/Al_2_O_3_  300 K, cycle 1 | 4 | 4.9×10^20^ | 18.5 |

Table S2. Summary of the material parameters extracted from the ellipsometry fit. When performing the ellipsometry fit we used the high-frequency permittivity $\varepsilon_{\infty}$, the electron density *N*, and the electron mobility *µ* as fitting parameters.


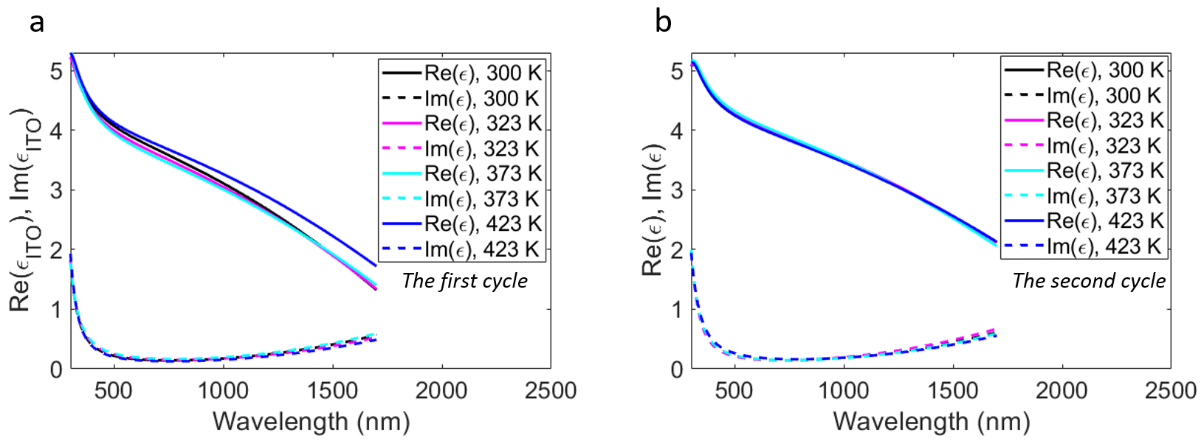


**Figure S14.** Ellipsometrically measured real (Re(ε_ITO_)) and imaginary (Im(ε_ITO_)) parts of the complex dielectric permittivity of a 16.7 nm-thick ITO film on a Si substrate. The ellipsometric measurements were taken at different temperatures indicated in the legends of a) and b). a) corresponds to the first temperature cycle while b) corresponds to the second temperature cycle.


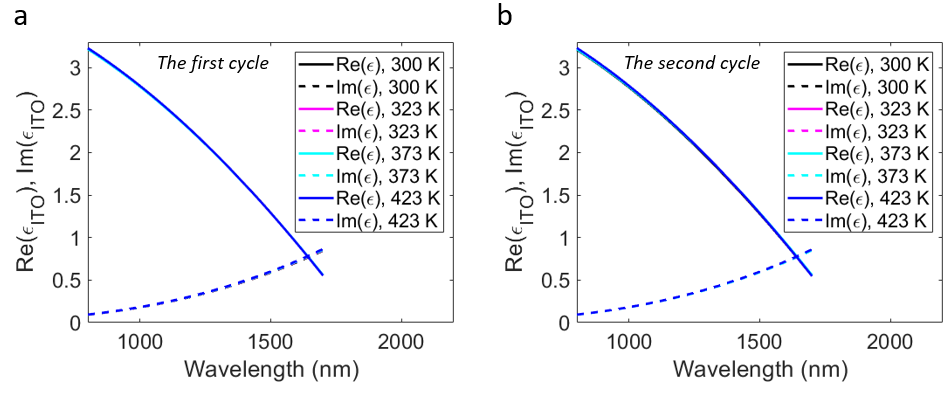


**Figure S15.** Ellipsometrically measured real (Re(ε_ITO_)) and imaginary (Im(ε_ITO_)) parts of the complex dielectric permittivity of a 16.7 nm-thick ITO film extracted from measuring Si/Au/ITO/Al_2_O_3_ heterostructure. The thicknesses of the layer in the heterostructure are as follows: Au – 80 nm, ITO – 16.7 nm, Al_2_O_3_ – 8.3 nm. The ellipsometric measurements were taken at different temperatures indicated in the legends of a) and b). a) corresponds to the first temperature cycle while b) corresponds to the second temperature cycle.

The observed trends are different when the ITO film is integrated into the Si/Au/ITO/Al_2_O_3_ heterostructure (Figure S14). In this case of the Si/Au/ITO/Al_2_O_3_ sample, ε_ITO_ does not vary noticeably with

increased temperature. We attribute the stark difference in the behavior of the sample d) (Si/ITO) and sample e) (Si/Au/ITO/Al_2_O_3_) to the presence of the capping Al_2_O_3_ layer in the sample e). To gain physical intuition, we fit the ellipsometry data only in the wavelength range 1000-1700 nm and use a single Drude model to describe the dielectric permittivity of ITO. The obtained fitting parameters are summarized in Table 2. The assumed effective electron mass is *m** = 0.35 *m_e_*. In case of the Si/ITO sample, Re(ε_ITO_) increases with temperature due to the decreased carrier density. Prior research has also shown that annealing ITO samples reduces the ITO carrier density.^20^ Additionally, we observe that the electron mobility reduces with increased temperature that is consistent with previous literature reports.^21^ Moreover, the electron mobility extracted from the measurements performed during the second temperature cycle is lower as compared with the values extracted during the first temperature cycle. This could be due to the defects introduced into ITO during the heating process.

Next, we discuss the properties of the ITO layer incorporated in the Si/Au/ITO/Al_2_O_3_ heterostructure (Figure S15). We observe that the extracted carrier density of the ITO is significantly higher as compared with that in the case when ITO has no capping layer (the Si/ITO sample). The observed increased carrier density is due to the oxygen leakage from the ITO layer during the Al_2_O_3_ ALD process. This result is consistent with previous literature reports^22^. It is also worth mentioning that during the ALD process the ITO film is effectively undergoing the annealing process since it is being heated to 423 K. The presence of the Al_2_O_3_ capping layer precludes the interaction of ITO with the ambient during the heating process. This explains the optical properties of the ITO layer in the Si/Au/ITO/Al_2_O_3_ heterostructure do not vary with increased temperature.

**9.** **Phase shift and reflectance modulation by CdO-based active metasurfaces**


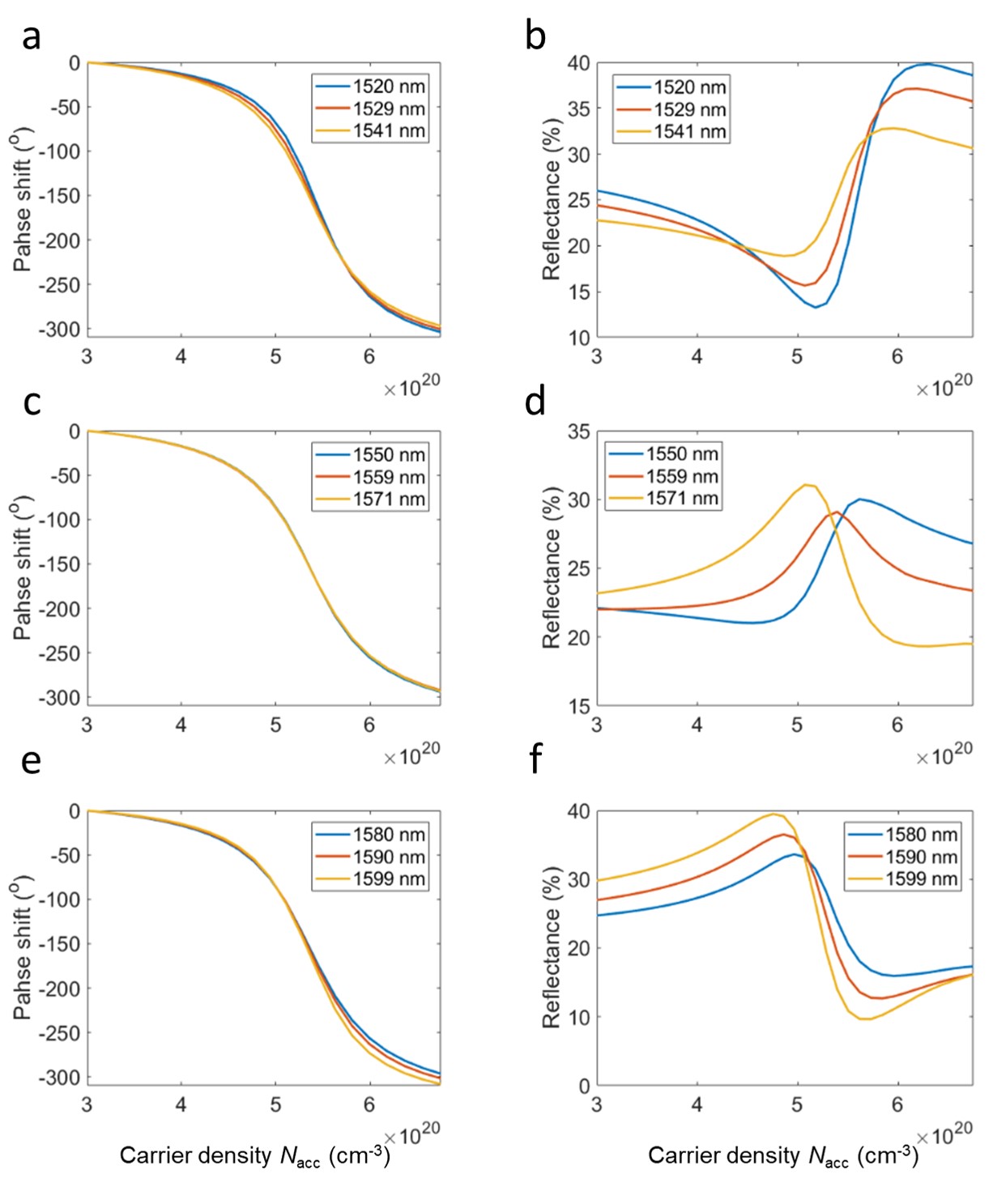


**Figure S16.** Electrically tunable optical response of the CdO-based gate-tunable metasurface. The geometrical parameters of our CdO-based gate-tunable metasurface are the same as the geometrical parameters of the ITO-based metasurface shown in Figure 1. The only difference between the ITO- and CdO-based metasurfaces is that we replaced the ITO layer in Figure 1a with a CdO layer. We plot the phase shift and the reflectance as a function of the carrier density of the active ITO layer for different fixed values of the operating wavelength. Legends indicate the values of the operating wavelengths in each case.

**10.** **Absorbance in the CdO-based active metasurfaces**


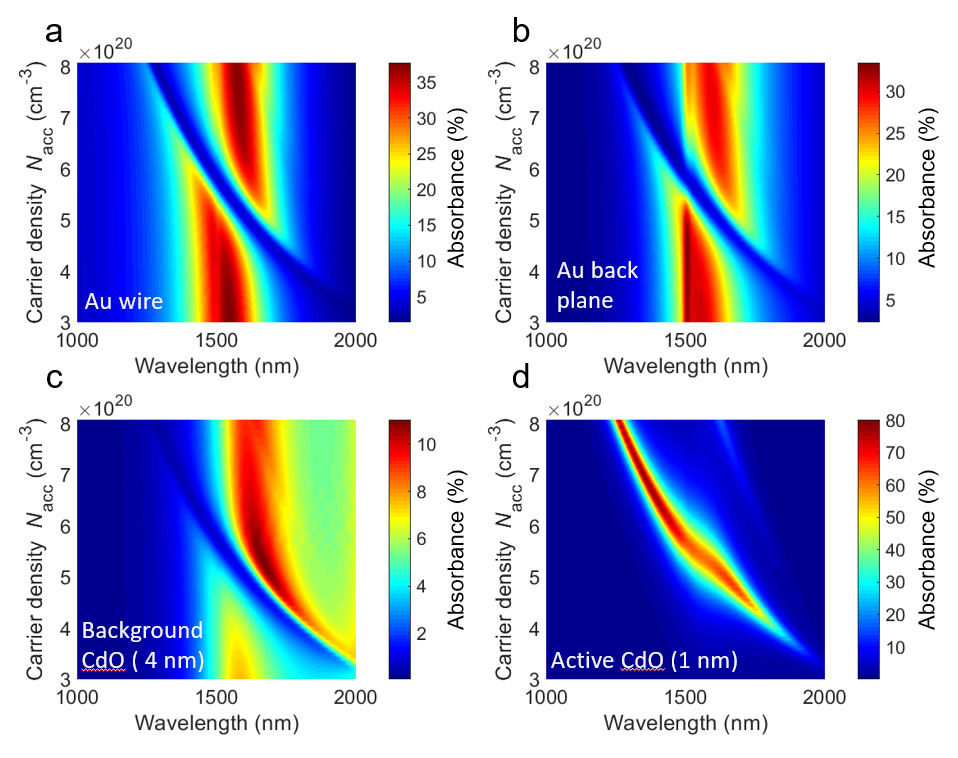


**Figure S17.** Absorbance in different constituent layers of the CdO-based active metasurface as a function of wavelength and carrier density in the active CdO layer. a) Absorbance in the Au wire. b) Absorbance in Au back plane. c) Absorbance in the 4 nm-thick non-modulated CdO layer. d) Absorbance in the 1 nm-thick active CdO layer.

**11.** **CdO-based active metasurfaces upon pulsed illumination and toxicity concerns**


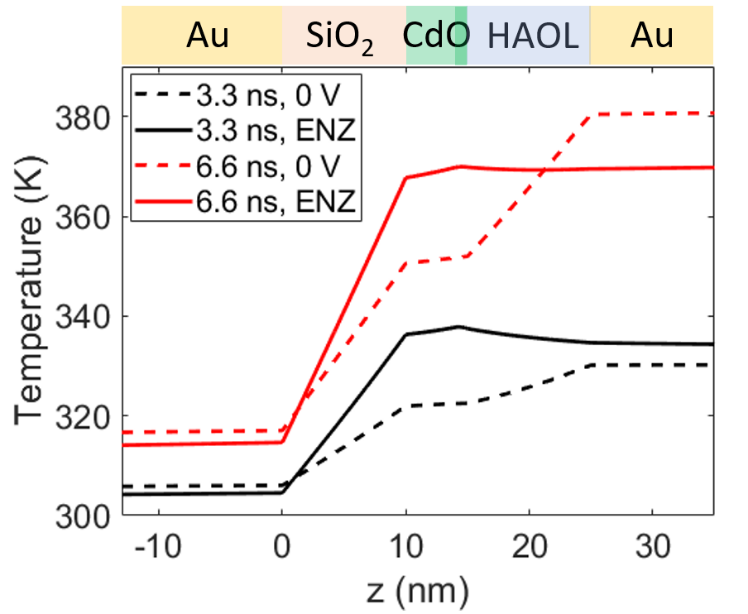


**Figure S18.** Temperature inside the metasurface element as a function of *z* coordinate at *x* =100 nm. Red and black curves correspond to the temperature distributions 3.3 ns and 6.6 ns after turning on the laser pulse, respectively. Dashed lines correspond to the case of no applied bias while solid lines correspond to the case when the CdO accumulation layer is in the ENZ regime (*N_acc_*=5.4×10^20^ cm^-3^). In case of the CdO- and ITO-based active metasurfaces, the illumination wavelengths are taken to be 1559 nm and 1550 nm, respectively.

We investigate the thermal performance of CdO-based gate-tunable metasurfaces in the case of pulsed laser illumination. The laser pulse shape and peak laser irradiance values are defined in Figure 5a and are the same as in the case of ITO-based metasurfaces. In the case of pulsed laser illumination, thermal performance of CdO-based metasurfaces exhibits multiple distinct features. When there is zero applied bias, compared with the Au wire temperature, we observe a visible temperature drop across both SiO_2_ and HAOL layers (Figure S18). When the CdO accumulation layer is in the ENZ regime, the temperature of the HAOL layer is equal or is slightly higher than the temperature of the Au wire. On the other hand, for ITO-based gate-tunable metasurfaces, we observed no temperature drop across the HAOL layer (Figure 4b). To understand why this temperature variation is observed, we re-examine resonant absorption in different layers of the CdO-based metasurface (Supporting Information, Part 10). Our calculations show that, with no applied bias, 38% of the incoming light is absorbed in the wire, 30% of the incoming light is absorbed in the back reflector, and only 9% of the incoming light is absorbed in the CdO. This contrasts significantly with the ITO-based gate-tunable metasurfaces for which the absorbance at resonant wavelengths is almost equally split between the back reflector, the ITO layer, and the Au wire
(Figure 1c). The high absorbance observed in the Au wire of the CdO-based metasurface results in a significantly elevated temperature of the Au wire when there is zero applied bias. In the ENZ regime, however, the CdO layer absorbs over 60% of the light, and the Au wire absorbs less than 10% of the light. Consequently, in the ENZ regime, CdO becomes the hottest layer of the metasurface at the beginning of the pulsed illumination (see Figure 5d, the plot corresponding to 3.3 ns). However, at later times (see Figure 5d, the plot corresponding to 6.6 ns), the ITO layer is almost at the same temperature as the Au wire. When comparing the overall thermal response of the ITO-based with the CdO-based metasurfaces in the transient illumination regime, we observe that the peak temperature attained in the ITO-based metasurfaces is higher (T_max_=400 K) than the peak temperature attained in the CdO-based metasurfaces (T_max_=380 K). Thus, as in the case of CW illumination, for a given illumination level, the CdO-based gate-tunable metasurface attains lower peak temperature than our ITO-based gate-tunable metasurface. The observed trends also indicate that unlike the case of steady-state illumination, in the pulsed illumination regime, the observed spatial temperature profile may significantly vary depending on the spatial distribution of absorption.

We use the described thermal analysis to estimate the performance of the CdO metasurface-based LiDAR and free space optical communications systems. The beam steering efficiency of the CdO-based active metasurface is ~22% because at a considered wavelength of 1559 nm the minimal reflectance of the metasurface is 22% (Figure 5b). Since Figure S1 assumes a beam steering efficiency of 22%, it can be used to evaluate the performance of the CdO metasurface-based LiDAR and free space optical communication systems. Note that for the assumed beam diameter of $D_{beam}=0.2$ cm, the incoming power of 0.1 kW corresponds to an irradiance of 3.2 kW/cm^2^. Our analysis shows that the irradiance of 3.2 kW/cm^2^ yields a temperature increase of 16 K when a 100 µm-thick Si substrate is assumed (Figure 5c). As seen in Figures S1c and S1e, in the case of incoming power of 0.1 kW, the LiDAR range of 220 m and optical communication distance of 100 km is attained. If the incoming CW power is 1 kW, the LiDAR range of 360 m and optical communication distance of 460 km can be attained but the temperature increase in the metasurface will be around 160 K. In case of the pulsed laser illumination, for the assumed beam diameter of $D_{beam}=0.2$ cm, the pulse energy of 0.1 mJ corresponds to the laser fluence of 3.2 mJ/cm^2^, which yields a maximal temperature increase slightly below 80 K. For the laser pulse energy of 0.1 mJ, the LiDAR range of 1 km and the optical communication distance of 8900 km is attained. These large communication distances and LiDAR ranges indicate that our CdO-based active metasurface is suitable for practical applications.

Finally, we would like to address a common concern regarding the toxicity of cadmium. Indeed, cadmium is a toxic heavy metal, and its usage is increasingly regulated. However, the use of cadmium is still permissible and manageable with proper controls, especially if it may offer a distinct advantage over alternatives. For example, HgCdTe amplified photodetectors can be purchased from Thorlabs since they are sensitive to light in the mid-infrared wavelength range. Analogously, even though arsenic is toxic, it is still used in photodetectors or solar cells powering aircrafts or spacecrafts. Thus, we conclude that even though cadmium is toxic, it can still be used in applications such as beam steering units for free space optical communications, for example.

Interestingly, cadmium is still widely used in the consumer products. European commission has practically banned the usage of cadmium in electrical and electronic products since it poses health and environmental risks when these electronic products are recycled.^23^ The corresponding directive of the European Union still provides a list of exceptions.^23^ Despite this, in the European union, new Ni-Cd batteries are still permitted but only in certain applications^24^. On the other hand, both in Europe and the United States, Ni-Cd batteries can readily be purchased in Amazon. Moreover, quite surprisingly, cadmium is widely used in brightly colored enameled drinking glasses as well as in decorated ceramic objects.^24^ Moreover, the thickness of cadmium layer in the proposed metasurface is only 5 nm, and it is covered by an additional HAOL layer. The final device will be coated by an additional protective layer further limiting the probability of cadmium exposure while handling the final device.

**12.** **Properties of the constituent materials**

|  | Density (kg/m^3^) | Specific heat (J/(kg×K)) | Thermal conductivity (W/(m×K)) | Electrical conductivity  (S/m) |
| --- | --- | --- | --- | --- |
| Au^25^ | 8933 | 385 | 100 | 4.005 ×10^7^ |
| Si^26^ | 2330 | 711 | 148 | 0.000311 |
| SiO_2_^27^ | 2203 | 709 | 0.8 | 0 |
| HAOL^28^ | 9680 | 261.28 | 1 | 0 |
| CdO^29-30^ | 8150 | 347.6 | 10 | 78700 |
| ITO^31^ | 7120 | 362.5 | 4 | 39000 |

Table S2. Summary of the material parameters in our thermal simulations.

The thermal properties of the HAOL films are assumed to be the same as the thermal properties of HfO_2_ films.^28^ In our optical simulations, HAOL and Al_2_O_3_ are modelled as non-dispersive materials with refractive indices of 2.07 and 1.745, respectively. Au was modelled by using the tabulated data by Palik.^32^

**13.** **The role of the thermal conductivity the thin SiO_2_ layer on the metasurface temperature: CW regime**

Here, we study how thermal conductivity of the SiO_2_ spacer layer between the Au back plane and the ITO layer affects the temperature of the metasurface. Let us consider the case of an infinite metasurface upon plane wave illumination. Consider the metasurface design shown in Figure 1, and replace the SiO_2_ with an imaginary material, which has the same refractive index as SiO_2_, but its thermal properties are identical to those of Au. This thought experiment allows us to understand the role of the thermal conductivities of the constituent materials without considering the variation in absorption density, which would occur if we changed the optical design of the metasurface. Assuming that the active ITO layer is in the ENZ regime, we then fix the value of the x coordinate to x = 100 nm and plot the temperature as a function of z coordinate (for definitions, see Figure 1a). Figure S18a corresponds to the case when thermal parameters of SiO_2_ are defined accurately, while in Figure S18b, thermal properties of SiO_2_ are identical to that of Au. As in Figure S18, the temperature values attained in both cases are very similar while the local temperature variation in Figure S18b is slightly more modest. We attribute the lack of dramatic differences between both cases (Figure S18b vs. Figure S18a) to the fact that the SiO_2_ layer is very thin, and, hence, despite low thermal conductivity, its thermal resistance is also quite modest.


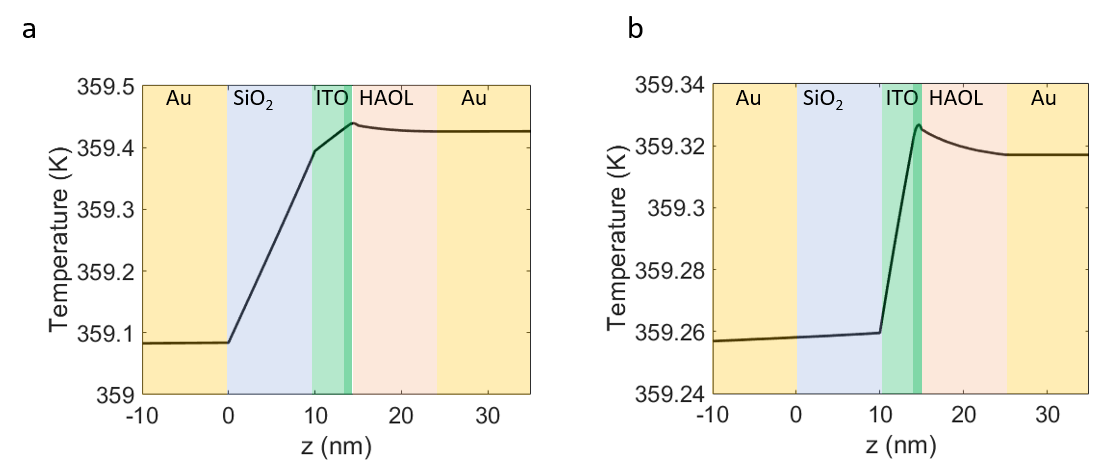
**Figure S19.** Temperature distribution inside the metasurface unit cell (see Figure 1a) when *x* is fixed as *x*=100 nm (dark green layer indicating the 1-nm–thick active accumulation layer). a) Thermal properties of SiO_2_ are taken into account accurately. b) Thermal properties of the SiO_2_ layer are replaced with those of Au. The substrate thickness is taken to be 250 μm, and we assume that the active ITO layer is in the ENZ regime. We also assume that we have a large metasurface shown in Figure 1a and use periodic boundary conditions.

**14.** **Characteristic time scales during pulsed laser illumination**

Next, we identify time scales over which our metasurface cools after being illuminated by a laser pulse (see Figure 2). The metasurface design and the specifics of the considered pulse are the same as in Figure 2 of the main manuscript. Thus, we extend our simulation time and calculate the evolution of the metasurface temperature over the time range from 0 to 3000 ns. Our simulation results are summarized in Figure S9. Figure S9 plots the maximal temperature of the metasurface during the pulsed laser illumination and after the pulse has been turned off. Note that the laser pulse is switched off at t=8.7 ns, which is marked by a vertical dashed line in Figure S9. We observe that 31 ns after the laser pulse has been turned off, the temperature increase of the metasurface is reduced by a factor of e^2^. Our simulations also show that 150 ns after the laser pulse has been turned on (or [150-8.7] ns after the laser pulse has been turned off), the temperature of the metasurface is reduced by a factor of e^3^. This indicates that the repetition rate of the pulsed laser used for illuminating metasurface can be ~6.7 MHz.


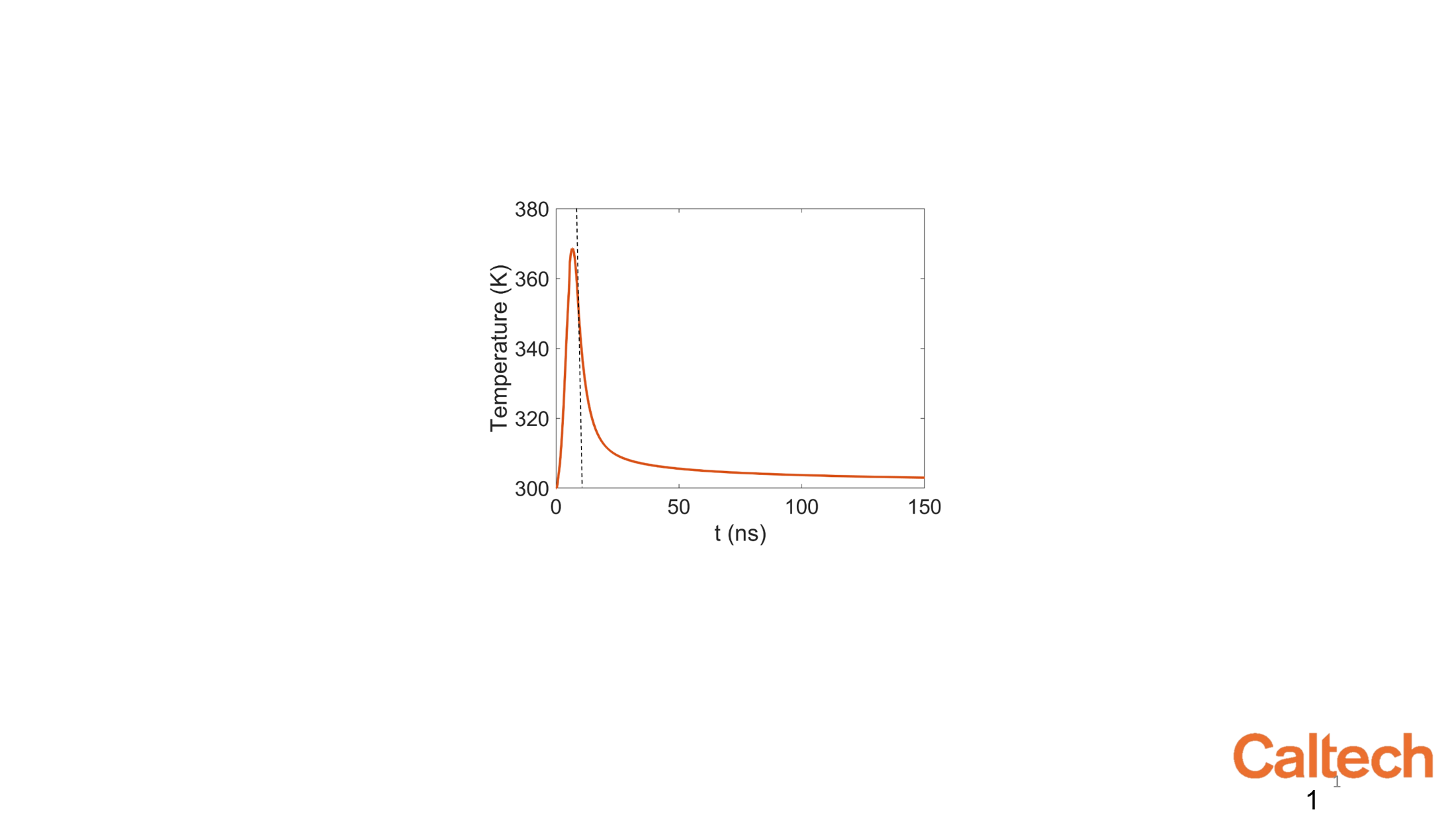


**Figure S20.** Maximal temperature of the metasurface under pulsed laser illumination as a function of time. The considered conditions are the same as in Figure 2 of the manuscript. The vertical dashed line marks the point in time when the pulse has been turned off.

**15.** **High-irradiance cycling and dynamic beam switching experiments**

Ideally, we would like to perform dynamic beam switching experiments upon high irradiance illumination.

However, for beam steering and phase measurements, a large area of the metasurface needs to be illuminated. Due to limited optical power at the output of the erbium doped fiber amplifier (EDFA), we are not able to perform direct beam steering and phase measurements upon high irradiance illumination. On the other hand, when performing reflectance measurements, we have been able to achieve high irradiances due to focusing of laser light down to the spot of 7 µm in diameter onto the sample.

Indeed, to perform beam switching experiments, we illuminate at least ten grating periods. In our current samples, 12 metasurface elements are electrically connected via a single electrode. Thus, for a two-level grating, the minimal grating period is 2 x 12 x 0.4 μm=9.6 μm (where 0.4 μm is the period of our metasurface). To observe switchable diffraction, we illuminate at least 10 grating periods, and, hence, our spot size is 100 μm in diameter. Because of this large required spot, we are not able to achieve high irradiances. Similarly, our phase shift measurement procedure requires illuminating the sample with a collimated beam and using the light reflected from the Au back plane as a reference to measure the shift of interference fringes. Thus, performing phase shift measurements upon focused light illumination is not optimal.

In summary, we performed the following measurements on the sample:

1. First, we performed reflectance, phase shift, and diffractive switching measurements upon low irradiance illumination when no amplification was applied. (Figures 5c and S21a, S21b).
2. Then we focused light down to a diameter of 7 µm and measured reflectance as a function of voltage at irradiance values of 1.6 kW/cm^2^, 4.9 kW/cm^2^, and 9.1 kW/cm^2^ (Figure 5d).
3. We repeated phase shift and switchable diffraction measurements (Figure S22).
4. We performed high-power cycling. In our cycling experiments, we select three distinct spots on the metasurface. We focused light onto each of the selected metasurface areas down to the spot of 7 µm in diameter and illuminated each spot with a laser beam, which has an irradiance of 9.1 kW/cm^2^. At each of the selected metasurface spots, we kept the focused light on for 10 s after which it was switched off for 10 s, and this sequence was repeated 20 times.
5. After performing high-power cycling, we remeasured the switchable diffraction upon low power illumination (~0.1 mW) (Figure S23).


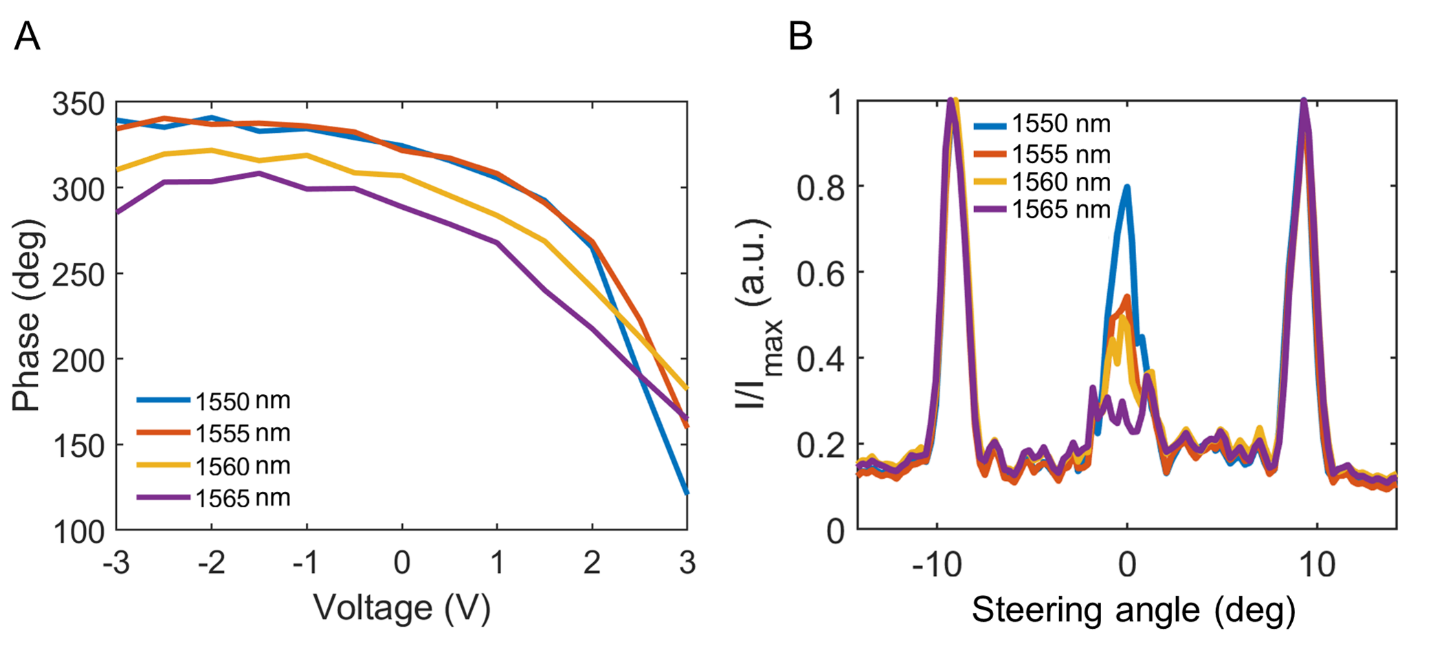


**Figure S21**. a) Measured phase as a function of voltage at low-irradiance illumination. b) Intensity of the electric field in the far field as a function of the steering angle. At no applied bias, a normally incident beam is specularly reflected (not shown here). When we apply 3 V to every other group of electrically connected metasurface elements and -3 V to adjacent electrically connected metasurface elements (see Figure 5a), we observe switchable diffraction. Both phase shift and switchable diffraction measurements are performed at multiple operating wavelengths specified in the legends of a) and b) (λ=1550 nm, λ=1555 nm, λ=1560 nm, λ=1565 nm). The measured values of the phase shift are as follows: 220° at λ=1550 nm, 181° at λ=1555 nm, 140° at λ=1560 nm, 144° at λ=1565 nm.


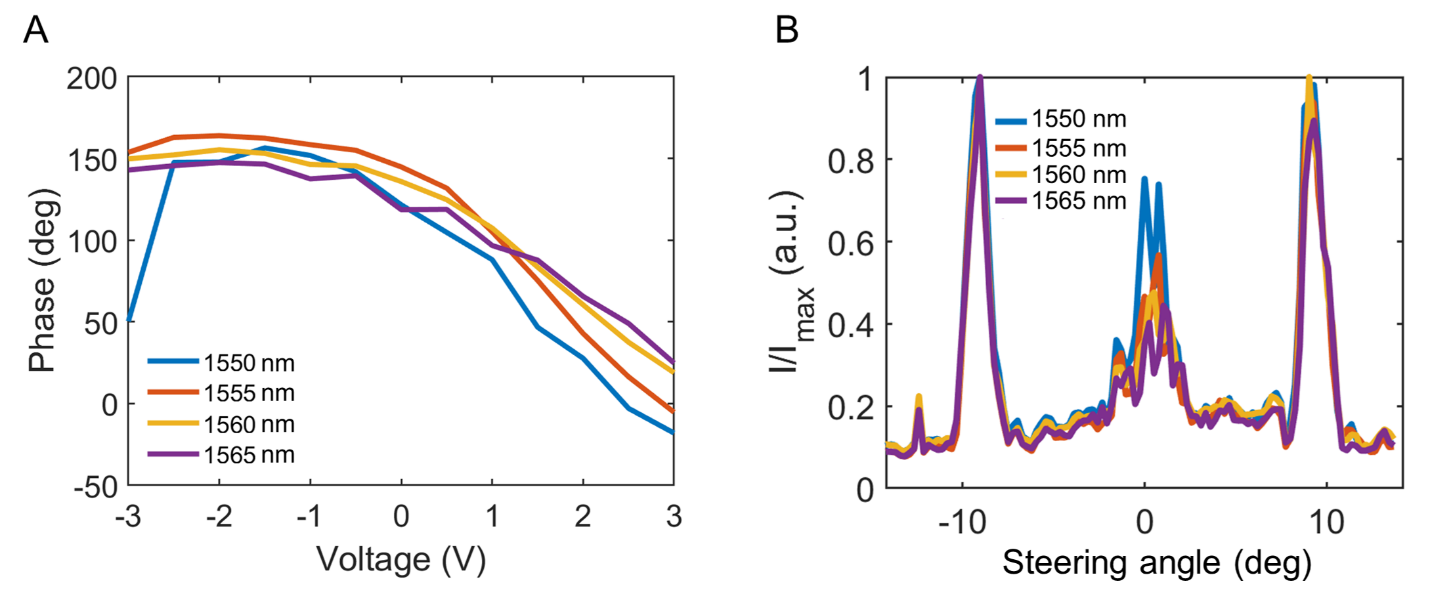


**Figure S22.** Measured phase a) and diffractive switching performance b) after high-irradiance measurements at one spot of the metasurface (see Fig. 5d). These experiments have been performed upon low irradiance (no amplification has been applied). b) Intensity of the electric field in the far field as a function of the steering angle. At no applied bias, a normally incident beam is specularly reflected (not shown here). When we apply 3 V to every other group of electrically connected metasurface elements and -3 V to adjacent electrically connected metasurface elements (see Figure 5a), we observe switchable diffraction. Both phase shift and switchable diffraction measurements are performed at multiple operating wavelengths specifies in the legends of a) and b) (λ=1550 nm, λ=1555 nm, λ=1560 nm, λ=1565 nm). In a), the measured values of the phase shift are as follows: 175° at λ=1550 nm, 169° at λ=1555 nm, 136° at λ=1560 nm, 123° at λ=1565 nm.


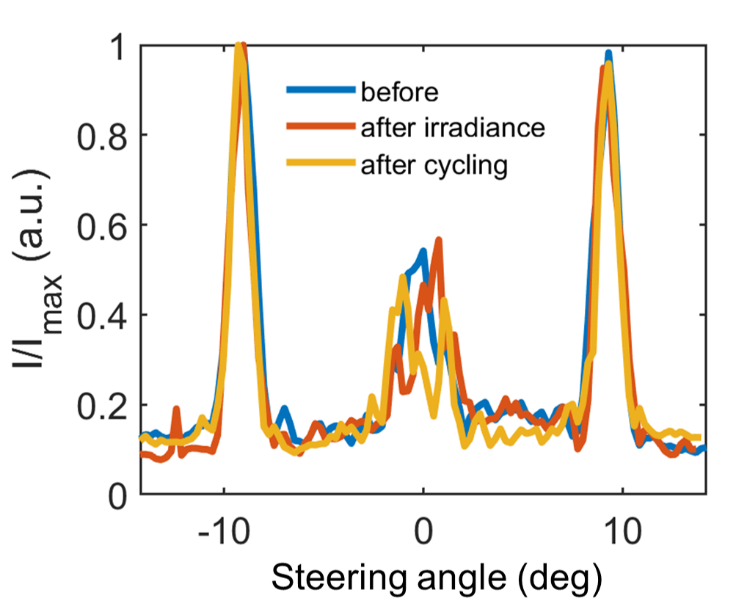


**Figure S23.** Intensity of the electric field in the far field as a function of the steering angle at an operating wavelength of λ=1555 nm. Blue curve has been measured before the sample has been subject to high-irradiance illumination. Red curve corresponds to the measurement taken after one spot on the metasurface has been illuminated with a high-irradiance beam and the measurement shown in Fig. 3 was taken. Yellow curve corresponds to the measurement taken after thermal cycling.

We observe that our metasurface exhibits a reflectance minimum at a wavelength of λ=1580 nm (Figure 5c). In our phase measurements, we observe that the largest phase shift (200°) is observed at a wavelength of λ=1550 nm (Figure S21a). To assess how high-irradiance illumination affects the optical response of the metasurface, we focused the CW laser beam onto the metasurface so that the diameter of the laser spot was 7 μm and measured the reflectance as a function of applied voltage (Figure 5d). After performing high-irradiance reflectance measurements, we re-measured gate-tunable phase shift and performed switchable diffraction measurements (Figure S22). We observed that the maximum achievable phase shift had been reduced (Figure S22a). The switchable diffraction pattern, on the other hand, did not noticeably change (Figure S22b). We have previously observed the reduction of the maximal achievable phase shift at each consecutive measurement that was reported in the Supplementary Information of our prior work ^1^. We hypothesized that applying bias to the metasurface degrades the gate dielectric giving rise to the pinholes. Based on this, we conclude that it is highly unlikely that the observed reduction of the phase shift is a result of the high irradiance illumination of the metasurface.

Next, we performed high-irradiance cycling on the metasurface. We selected three distinct areas on the metasurface and illuminated these areas with a focused laser beam with a maximal irradiance of 9.1 kW/cm^2^. Then we blocked (for 10 s) and unblocked (for 10 s) the light path with an opaque object 20 times, thus performing 20 heat/cool cycles at each of the selected areas. Next, we used the metasurface sample to demonstrate switchable diffraction by using a two-level phase grating approach at an operating wavelength of λ=1555 nm and at low irradiance when no amplification has been applied (Figure S23). We observed that thermal cycling did not noticeably affect the diffraction pattern indicating that our metasurface is robust with respect to high-irradiance illumination.

We have also acquired scanning electron microscopy (SEM) images after the metasurface has been illuminated (details of high-irradiance cycling are described in our response to question 1). We observe no visible signs of damage in the SEM images (Figures S24 and S25). When acquiring SEM images, we took closer look at the metasurface areas, which were used for gate-tunable reflectance and high-irradiance cycling measurements. We observed no detectable damage to the metasurface in none of these areas (Figures S24 and S25).


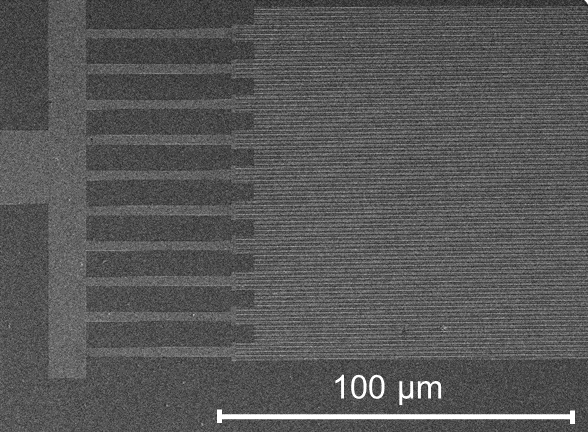


**Figure S24**. SEM image of the metasurface. Scale bar is 50 µm. We observe no visible damage on the metasurface.


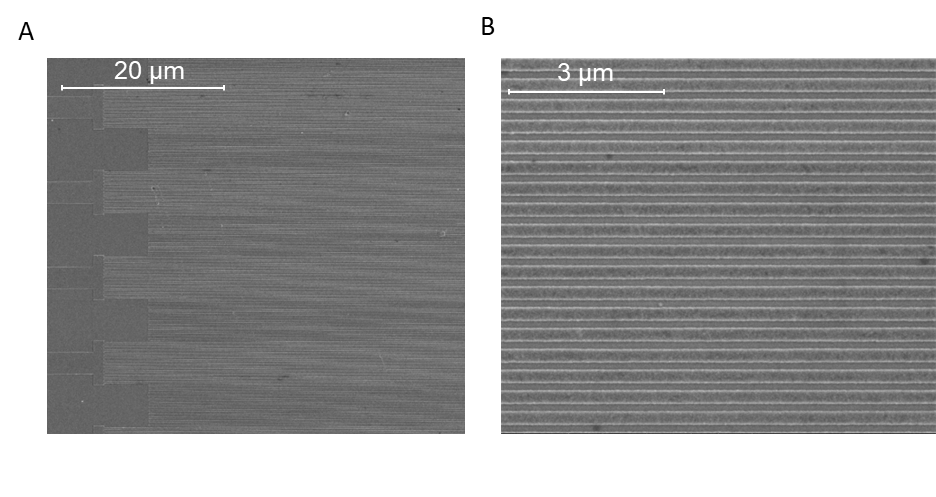


**Figure S25**. SEM images of the metasurface. In a), the scale bar is 20 µm. In b), the scale bar is 3 µm. We observe no visible damage on the metasurface.

AUTHOR INFORMATION

Corresponding Author:

Harry A. Atwater

^*^E-mail: [haa@caltech.edu](mailto:haa@caltech.edu)

Present Address

†Seurat Technologies, Inc., 265 Ballardvale St, Wilmington, MA 01887, USA

**References**

1. McManamon, P., *Lidar Range Equation*. Springer: 2015.

2. Clymer, D. R.; Cagan, J.; Beuth, J., Power–Velocity Process Design Charts for Powder Bed Additive Manufacturing. *Journal of Mechanical Design* **2017,** *139* (10).

3. Amin, R.; Maiti, R.; Gui, Y.; Miscuglio, M.; Heidari, E.; Chen, R. T.; Dalir, H.; Sorger, V. J. In *Broadband GHz ITO-Based Plasmon MZI Modulator on Silicon Photonics*, Conference on Lasers and Electro-Optics, Washington, DC, 2020/05/10; Optical Society of America: Washington, DC, 2020; p FM2R.2.

4. Kafaie Shirmanesh, G.; Sokhoyan, R.; Pala, R. A.; Atwater, H. A., Dual-Gated Active Metasurface at 1550 nm with Wide (>300 Degrees) Phase Tunability. *Nano Lett* **2018,** *18* (5), 2957-2963.

5. Huang, Y. W.; Lee, H. W.; Sokhoyan, R.; Pala, R. A.; Thyagarajan, K.; Han, S.; Tsai, D. P.; Atwater, H. A., Gate-Tunable Conducting Oxide Metasurfaces. *Nano Lett* **2016,** *16* (9), 5319-25.

6. Reines, I. C.; Wood, M. G.; Luk, T. S.; Serkland, D. K.; Campione, S., Compact Epsilon-Near-Zero Silicon Photonic Phase Modulators. *Opt Express* **2018,** *26* (17), 21594-21605.

7. Vermeersch, B.; De Mey, G., Influence of Substrate Thickness on Thermal Impedance of Microelectronic Structures. *Microelectronics Reliability* **2007,** *47* (2), 437-443.

8. Darwish, A. M.; Hung, H. A.; Bayba, A. J.; El-Kinawi, K., Accurate Calculation of Junction Temperature of HBTs. *IEEE Transactions on Microwave Theory and Techniques* **2011,** *59* (3), 652-659.

9. Coppens, Z. J.; Li, W.; Walker, D. G.; Valentine, J. G., Probing and Controlling Photothermal Heat Generation in Plasmonic Nanostructures. *Nano Letters* **2013,** *13* (3), 1023-1028.

10. Zolotavin, P.; Alabastri, A.; Nordlander, P.; Natelson, D., Plasmonic Heating in Au Nanowires at Low Temperatures: The Role of Thermal Boundary Resistance. *ACS Nano* **2016,** *10* (7), 6972-6979.

11. Elhadj, S.; Matthews, M. J.; Yang, S. T.; Cooke, D. J.; Stolken, J. S.; Vignes, R. M.; Draggoo, V. G.; Bisson, S. E., Determination of the Intrinsic Temperature Dependent Thermal Conductivity from Analysis of Surface Temperature of Laser Irradiated Materials. *Applied Physics Letters* **2010,** *96* (7), 071110.

12. Tumkur, T. U.; Sokhoyan, R.; Su, M. P.; Ceballos-Sanchez, A.; Kafaie Shirmanesh, G.; Kim, Y.; Atwater, H. A.; Feigenbaum, E.; Elhadj, S., Toward High Laser Power Beam Manipulation with Nanophotonic Materials: Evaluating Thin Film Damage Performance. *Opt. Express* **2021,** *29* (5), 7261-7275.

13. Neudeck, P. G.; Okojie, R. S.; Liang-Yu, C., High-Temperature Electronics - a Role for Wide Bandgap Semiconductors? *Proceedings of the IEEE* **2002,** *90* (6), 1065-1076.

14. Huang, Y.-W.; Lee, H. W. H.; Sokhoyan, R.; Pala, R. A.; Thyagarajan, K.; Han, S.; Tsai, D. P.; Atwater, H. A., Gate-Tunable Conducting Oxide Metasurfaces. *Nano Letters* **2016,** *16* (9), 5319-5325.

15. Shirmanesh, G. K.; Sokhoyan, R.; Wu, P. C.; Atwater, H. A., Electro-Optically Tunable Multifunctional Metasurfaces. *ACS Nano* **2020,** *14* (6), 6912-6920.

16. Incropera, F. P., *Introduction to heat transfer*. 5th ed.; Wiley: Hobokenm NJ, 2007; p xxv, 901 p.

17. Reddy, H.; Guler, U.; Kildishev, A. V.; Boltasseva, A.; Shalaev, V. M., Temperature-Dependent Optical Properties of Gold Thin Films. *Opt. Mater. Express* **2016,** *6* (9), 2776-2802.

18. Lee, S., Song, S., Au, V., and Moran, K.P, Constriction/Spreading Resistance Model for Electronic Packaging. *Proceedings of ASME/JSME Engineering Conference* **1995,** *4*.

19. Gong, J.; Dai, R.; Wang, Z.; Zhang, C.; Yuan, X.; Zhang, Z., Temperature Dependent Optical Constants for SiO_2_ Film on Si Substrate by Ellipsometry. *Materials Research Express* **2017,** *4* (8), 085005.

20. Wu, C.-Y.; Thanh, T. V.; Chen, Y.-F.; Lee, J.-K.; Lin, J.-J., Free-Electronlike Diffusive Thermopower of Indium Tin Oxide Thin Films. *Journal of Applied Physics* **2010,** *108* (12), 123708.

21. Trier, F.; Christensen, D. V.; Pryds, N., Electron Mobility in Oxide Heterostructures. *Journal of Physics D: Applied Physics* **2018,** *51* (29), 293002.

22. Bayer, T. J. M.; Wachau, A.; Fuchs, A.; Deuermeier, J.; Klein, A., Atomic Layer Deposition of Al2O3 onto Sn-Doped In2O3: Absence of Self-Limited Adsorption during Initial Growth by Oxygen Diffusion from the Substrate and Band Offset Modification by Fermi Level Pinning in Al2O3. *Chemistry of Materials* **2012,** *24* (23), 4503-4510.

23. Directive 2002/95/EC on the Restriction of the Use of Certain Hazardous Substances in Electrical and Electronic Equipment. *Off. J. Eur. Union* **2003,** *L37* (19).

24. Turner, A., Cadmium Pigments in Consumer Products and Their Health Risks. *Science of The Total Environment* **2019,** *657*, 1409-1418.

25. Linseis, V.; Völklein, F.; H.Reith; K.Nielsch; P.Woiasd, Thermoelectric Properties of Au and Ti Nanofilms, Characterized with a Novel Measurement Platform. *Materials Today: PROCEEDINGS* **2019,** *8*, 517-522.

26. Plawsky, J. L., *Transport Phenomena Fundamentals*. Third edition. ed.; CRC Press, Taylor & Francis Group: Boca Raton, 2014.

27. Kato, R.; Hatta, I., Thermal Conductivity Measurement of Thermally-Oxidized SiO2 Films on a Silicon Wafer Using a Thermo-Reflectance Technique. *International Journal of Thermophysics* **2005,** *26*, 179–190.

28. Scott, E. A.; Gaskins, J. T.; King, S. W.; Hopkins, P. E., Thermal Conductivity and Thermal Boundary Resistance of Atomic Layer Deposited High-k Dielectric Aluminum Oxide, Hafnium Oxide, and Titanium Oxide Thin Films on Silicon. *APL Materials* **2018,** *6* (5), 058302.

29. Donovan, B. F.; Sachet, E.; Maria, J.-P.; Hopkins, P. E., Interplay Between Mass-Impurity and Vacancy Phonon Scattering Effects on the Thermal Conductivity of Doped Cadmium Oxide. *Applied Physics Letters* **2016,** *108* (2), 021901.

30. Madelung, O.; Rössler, U.; Schulz, M., *Semiconductors · II-VI and I-VII Compounds; Semimagnetic Compounds*. Springer: 1999.

31. Ashida, T.; Miyamura, A.; Oka, N.; Sato, Y.; Yagi, T.; Taketoshi, N.; Baba, T.; Shigesato, Y., Thermal Transport Properties of Polycrystalline Tin-Doped Indium Oxide Films. *Journal of Applied Physcis* **2009,** *105*, 073709.

32. Palik, E. D.; Ghosh, G., *Handbook of Optical Constants of Solids*. Academic Press: San Diego, 1998.
